# Molecular systematics of *Keratinophyton*: the inclusion of species formerly referred to *Chrysosporium* and description of four new species

**DOI:** 10.1186/s43008-021-00070-2

**Published:** 2021-07-08

**Authors:** Roman Labuda, Andreas Bernreiter, Doris Hochenauer, Alena Kubátová, Hazal Kandemir, Christoph Schüller

**Affiliations:** 1 Department for Farm Animals and Veterinary Public Health, Institute of Food Safety, Food Technology and Veterinary Public Health; Unit of Food Microbiology, University of Veterinary Medicine Vienna, Veterinaerplatz 1, 1210 Vienna, Austria; 2 Research Platform Bioactive Microbial Metabolites (BiMM), Konrad Lorenz Strasse 24, 3430 Tulln a.d. Donau, Austria; 3 Charles University, Faculty of Science, Department of Botany, Culture Collection of Fungi (CCF), Benátská 2, 128 01 Prague 2, Czech Republic; 4 Center of Expertise in Mycology of Radboud University Medical Centre, Canisius Wilhelmina Hospital, Geert Grooteplein Zuid 10, 6525GA Nijmegen, The Netherlands; 5 Division of Mycology, Faculty of Medicine, Çukurova University, Balcalı 01330, Sarıçam, Adana Turkey; 6 Core Facility Bioactive Molecules Screening and Analysis and Institute of Microbial Genetics, University of Natural Resources and Life Sciences, Vienna (BOKU), Konrad Lorenz Strasse 24, 3430 Tulln a.d. Donau, Austria

**Keywords:** *Chrysosporium*, Keratinophilic fungi, Keratinolysis, One fungus = one name, New taxa

## Abstract

Four new *Keratinophyton* species (*Ascomycota*, *Pezizomycotina*, *Onygenales*), *K. gollerae*, *K. lemmensii*, *K. straussii,* and *K. wagneri*, isolated from soil samples originating from Europe (Austria, Italy, and Slovakia) are described and illustrated. The new taxa are well supported by phylogenetic analysis of the internal transcribed spacer region (ITS) region, the combined data analysis of ITS and the nuclear large subunit (LSU) rDNA, and their phenotype. Based on ITS phylogeny, within the *Keratinophyton* clade, *K*. *lemmensii* is clustered with *K*. *durum, K*. *hubeiense*, *K*. *submersum,* and *K*. *siglerae,* while *K*. *gollerae*, *K*. *straussii* and *K*. *wagneri* are resolved in a separate terminal cluster. All four new species can be well distinguished from other species in the genus based on phenotype characteristics alone. Ten new combinations are proposed for *Chrysosporium* species which are resolved in the monophyletic *Keratinophyton* clade. A new key to the recognized species is provided herein.

**Supplementary Information:**

The online version contains supplementary material available at 10.1186/s43008-021-00070-2.

## INTRODUCTION

*Keratinophyton* is a genus of microscopic fungi (*Ascomycota*, *Onygenales*, *Onygenaceae*) comprising species that live mostly on the remains of hair and feather in soil as saprotrophs (Cano and Guarro 1990; Hubka et al. 2016; Sutton et al. 2013; Vidal et al. 2000). Formerly, they were classified in *Aphanoascus* mainly based on the presence of ascomata (cleistothecia) composed of a membranous peridium (Cano and Guarro 1990; Cano et al. 2002). In a review employing a phenotypic and phylogenetic approach, Cano et al. (2002) accepted 18 *Aphanoascus* species which all have sexual morphs. Only recently, the polyphyletic status of *Aphanoascus s. lat.* has been resolved by Sutton et al. (2013) who established the genus *Keratinophyton* encompassing and redisposing six species, namely *K*. *durum, K*. *hispanicum, K*. *multisporum, K*. *punsolae, K*. *saturnoideum* and the type species *K*. *terreum*. Ascospores of *Keratinophyton* species are characterized by a conspicuous equatorial rim and pitted wall, while *Aphanoascus* species have reticulate ascospores without a rim (Sutton et al. 2013). Within *Keratinophyton,* only *K*. *multiporum* is related to a *Malbranchea* asexual morph (Cano and Guarro 1990), while the remaining known species have a *Chrysosporium* asexual morph. In addition to the above mentioned species, the monophyletic *Keratinophyton* clade currently encompasses at least 11 species known only as asexual morphs (Cano and Guarro 1990; Hubka et al. 2016; Sharma and Shouche 2017; Liang et al. 2009; van Oorschot 1980; Vidal et al. 2000; Vidal et al. 2002; Zhang et al. 2016; Zhang et al. 2017). Sharma and Shouche (2017) introduced a new species, *Keratinophyton turgidum,* based on the morphology of its chrysosporium-like aleurioconidia and ITS locus phylogeny. The same authors stated that all species in this monophyletic clade which have a *Chrysosporium* asexual morph require redisposing in the genus *Keratinophyton*.

The presence of this large group of ubiquitous and keratinolytic species is rather common especially in areas with high animal activity that results in transfer of the keratinous material (fur, hairs, etc.) to the soil (Papini et al. 1998; Vidal et al. 2000). The following reports confirm their world-wide distribution and occurrence in different habitats usually associated with soil environments, e.g. soil in city parks (Papini et al. 1998; Vidyasagar et al. 2005), flower pots (Singh et al. 2009), sand in children’s sandpits (Labuda et al. 2008), mud (Zaki et al. 2005), poultry farms (Anbu et al. 2004; Cano and Guarro 1990), marshy meadows, salt pans, desert, cultivated or uncultivated soils (Cano and Guarro 1990; Chmel and Vláčilíková 1977; Deshmukh 2004; Deshmukh et al. 2008; Han et al. 2013; Javorekova et al. 2012; Zhang et al. 2016; Zhang et al. 2017) and river sediments (Ulfig et al. 1997; Vidal et al. 2000; Vidal et al. 2002). In general, these fungi are rarely reported as animal pathogens, and in fact, only two species *C*. *echinulatum* and *C*. *pannicola* (formerly known as *C*. *evolceanui*) have been involved in mycoses (Hajsig et al. 1974; Cabanes et al. 2014; Hubka et al. 2016).

During a microbiological survey of environmental samples (soil and compost) in July 2019, several interesting *Chrysosporium* asexual morphs were isolated. These isolates were phenotypically similar to those previously isolated from the same samples in August 2015 by one us (R.L.). These isolates were designated BiMM-F76, BiMM-F77 (also strain RL-07, isolated in July 2019), BiMM-F78 (also strains RL-05 and RL-06, isolated in July 2019), and BiMM-F250. All strains were further characterized in terms of morphology, physiology, and molecular phylogeny. Phylogenetically informative sequences were obtained from the internal transcribed spacer (ITS) region and the nuclear large subunit (LSU) rDNA. Overall, the resulting data revealed that these isolates represent novel species of the genus *Keratinophyton*, and they were illustrated for the first time in this paper.

## MATERIALS AND METHODS

### Sample collection and isolation of the fungi

A sample of a garden soil in Vieste (Italy) was collected in July 2004, one of a forest soil in Tatranská Lomnica (The Slovak Republic) in August 2011, and one of compost from an agricultural base at the Institute of Agrobiotechnology (IFA Tulln, Austria) in August 2015. All three samples were taken from the surface layer (3–5 cm deep), dried, and stored in plastic bags in a fridge (5–8 °C) until the time of analysis (August 2015 and July 2019). Isolation of the keratinophilic fungi was performed as described previously (Javoreková et al. 2012). Each sample was divided into 10 subsamples. The subsamples (20 g each) were poured into Petri dishes and soaked with antibiotic solution containing 0.5 g cycloheximide and 0.1 g chloramphenicol. Sterile defatted horse hair fragments (10 pieces of ca 2.0 cm per plate) were used as baits. The Petri dishes were then incubated at laboratory temperature (23–25 ± 1 °C), under ambient daylight, for a period of 2–3 months and remoistened with sterile deionized water when necessary. The Petri dishes were checked weekly for the presence of fungi, and isolates were cultured on Sabouraud 4% dextrose agar (SDA; Merck, Darmstadt, Germany) supplemented with 0.5 g cycloheximide and 0.05 g chloramphenicol. Pure cultures were then transferred onto potato dextrose agar [PDA; Van Waters and Rogers (VWR) International, Leuven, Belgium]. The preliminary identification of the resulting keratinophilic fungi was carried out based on their phenotypic characteristics according to van Oorschot (1980) and Vidal et al. (2000, 2002).

### Morphological analysis

For phenotypic determination, the strains were transferred (three-point inoculation with a needle) to PDA, Malt Extract Agar (MEA; Merck, Darmstadt, Germany), and SDA, and incubated for 14 d in the dark at 25 °C. Christensen’s urea agar (Sigma-Aldrich, St Louis, MO, USA) was used for additional physiological and biochemical characteristics (25 °C, 14 d, in the dark). Corn Meal Agar (CMA; Oxoid, Basingstoke, UK), Potato Carrot Agar (PCA) (Samson et al. 2010) and Emerson YpSs agar (Atlas 1946) were used for stimulation of sexual reproduction (at 20 °, 25 °, and 28 °C, for up to 3 months in the dark).

Colony size (mm), colony structure and characteristics were noted after 14 d (on PDA, MEA, SDA, PYE, YpSs, CMA, and PCA). However, the cultivation was extended up to 3 months to observe and record changes in pigmentation of the colonies as well as to determine the onset of sexual reproduction. In order to determine the optimal and minimum/maximum temperatures for growth, PDA, MEA and SDA plates were incubated at 5 °, 8 °, 10 °, 12 °, 15 °, 18 °, 20 °, 25 °, 28–32 °, 35 °, and 37 °C, and the growth rate was measured on the 14th day of cultivation. For comparative descriptions of the macroscopic and microscopic characteristics, PDA was used according to Vidal et al. (2002), Hubka et al. (2016) and Sharma and Shouche (2017).

For determination of microscopic traits, PDA was used after 14–18 d. Conidiophore and conidia formation were observed in situ under low magnification (50–100x). Details of conidiophores, conidia (aleurioconidia) and other microscopic structures, such as width of hyphae, were observed in Melzer’s reagent and lactic acid with cotton blue. Photomicrographs were taken in Melzer’s reagent and lactic acid with cotton blue using phase and Nomarski contrast optics on an Olympus BX51 microscope with Olympus DP72 camera and QuickPHOTO Micro 3.0 software. Photographs of the colonies were taken with a Sony DSC-RX100.

Scanning electron microscopy (SEM) was performed on a JEOL JSM-6380 LV microscope (JEOL, Tokyo, Japan). Fungal samples were prepared according to a simplified method (Samson et al. 1979). Pieces of colonies (ca. 3 × 5 mm) growing on PDA were fixed in 6% glutaraldehyde overnight in the refrigerator (ca. 20 h), then dehydrated in 2-methoxyethanol for 10 min. This was followed by critical point drying and gold coating in a BAL-TEC SCD 050 Sputter Coater. The samples were observed with spot size 35–39 and accelerating voltage 20–23 kV.

Dried fungarium specimens deposited as holotypes in the collections of the Mycological Department, National Museum in Prague, Czech Republic (PRM); ex-type cultures were deposited in the Bioactive Microbial Metabolites (BiMM) Fungal Collection, UFT- Tulln in Austria and in the Culture Collection of Fungi in Prague (CCF).

### Keratinolytic activity

Keratinolytic activity was tested by placing a few sterilized blond hairs of a 5 y old child on a PDA plate 1 cm away from the point of inoculation (van Oorschot 1980). Ability to digest keratin was observed after 21 d of incubation at 25 °C in the dark. In addition, a hair perforation test was also performed following de Hoog et al. (2020) using 25 mL water containing 2–3 drops 10% yeast extract (YEW). The hairs were examined microscopically after 14 and 21 d of the inoculation at 25 °C in the dark. At the end of the incubation period, a few pieces of hair were taken out from the testing media (PDA and YEW). The overgrowing fungus was deactivated with 70% ethanol and then removed from the hair surface mechanically in a stream of a tap water. The degree of hair digestion-degradation (keratinolytic activity) was assessed in the light microscope under 100x and 400x magnification. For the observation and microphotography of the hairs, water was used as mounting fluid. Intensity of degradation of the hair was estimated on a scale of 0 to 4 (Marchisio et al. 1994): 0 = no degradation; 0–1 = light degradation on the cuticle; 1 = moderate degradation on the cuticle and/or rare formation of boring hyphae; 2 = degradation of cuticle and cortex, with about 20% degradation of the hair; 3 = degradation of cuticle and cortex, with about 50% degradation of the hair; 4 = degradation of cuticle and cortex, with about 80% degradation of the hair. The photomicrographs of the hairs were taken using a Motic BA 310 microscope with Motic Image Plus 3.0 software. The final microscopic pictures were black-and-white inverted.

### DNA extraction, PCR amplification and sequencing

DNA was extracted using a standard cetyltrimethyl ammonium bromide (CTAB) procedure, as described previously (Doyle and Doyle 1987). The internal transcribed spacer (ITS) region was amplified with primers ITS1-F (Gardes and Bruns 1993) and ITS4 (White et al. 1990) using Taq-polymerase. The D1*/*D2 domains of the large-subunit (28S) rRNA gene (LSU) were amplified and sequenced using the primer pair ITS1/TW14 (White et al. 1990; Mori et al. 2000). All reactions were performed in an Eppendorf Gradient MasterCycler (Eppendorf, Hamburg, Germany). Conditions for amplification of ITS and LSU domains: 95 °C for 5 min; 35 cycles of 95 °C for 30 s, 54 °C for 30 s, 72 °C for 90 s, and finally 5 min at 72 °C. The PCR products were sequenced with the same primers used for the PCR amplifications (Microsynth, Balgach, Switzerland). All sequences obtained in this study were deposited in GenBank nucleotide database (Table 1).

**Table 1:** List of the strains included in the study

Species name	Strain^a^	Source	GenBank accession numbers	
			ITS	LSU
*A. canadensis*	UAMH 4574	Carnivore dung, Canada	AJ439435	–
*A. clathratus*	IMI 329400	Arable soil, Spain	AJ439436	–
*A. cubensis*	FMR 4220	Soil of tobacco field, Cuba	AJ439432	–
*A. foetidus*	CBS 453.75^T^	*Myomys daltoni* coat, Nigeria	KT155907	KT155252
*A. fulvescens*	NBRC 30411	Soil of rice paddy field, Japan	JN943432	JN941547
*A*. *keratinophilus*	IFM 55159^T^	Pasture land soil, Papua New Guinea	NR165936	NG064030
*A*. *mephitalis*	IMI 151084^T^	Dung of wolf, Canada	AJ439439	AY176725
*A*. *orissae*	CBS 340.89	Soil in animal husbandry, Kuwait	AJ390393	–
*A*. *pinarensis*	FMR 4221	Forest soil, Cuba	AJ439433	–
*A*. *reticulisporus*	CBS 392.67^T^	Soil, New Zealand	MH859002	MH870704
*A*. *verrucosus*	NBRC 32381^T^	Arable soil, Spain	NR131309	NG057011
*K*. *clavisporum* (*C*. *clavisporum*)	G80.1^T^	Plant root soil, China	KY026601	–
*K*. *durum*	CBS 118.85^T^	Soil, Nepal	MH861856	AB075345
*K*. *echinulatum* (*C*. *echinulatum*)	CCF 4652 ^T^	Sole of the foot, Czechia	LT548276	LT548276
*K*. *fluviale* (*C*. *fluviale*)	FMR 6005^T^	River sediments, Spain	AJ005367	**MT875000**
***K*.** ***gollerae***	**BiMM F250**^T^	**Forest soil, Slovakia**	**MN633084**	**MT874997**
*K*. *hispanicum*	CBS 456.90^T^	Beach soil, Spain	KT155910	**MT875003**
*K*. *hubeiense* (*C*. *hubeiense*)	EM66601^T^	Soil under the chicken feather, China	KJ849227	–
***K*.** ***lemmensii***	**BiMM F76**^T^	**Compost soil, Austria**	**MN633082**	**MT874998**
*K*. *linfenense* (*C*. *linfenense*)	GZAC H31^T^	Rhizosphere soil, China	NR158289	–
*K*. *minutisporosum* (*C*. *minutisporosum*)	IMI 379912^T^	River sediments, Spain	KT155616	**MT875001**
*K*. *pannicola* (*C*. *pannicola*)	CBS 116.63^T^	Soil, India	AJ005368	MH869834
*K*. *punsolae*	IMI 334818^T^	Arable soil, Spain	AJ439440	–
*K*. *qinghaiense* (*C*. *qinghaiense*)	GZUIFR Chry 11^T^	Farmland soil, China	JX868607	–
*K*. *saturnoideum*	CBS 628.88^T^	Arable soil, Spain	NR077135	AB075347
*K*. *siglerae* (*C*. *siglerae*)	UAMH 6541^T^	Garden soil, Spain	AJ131684	**MT875002**
***K*.** ***straussii***	**BiMM F78**^T^	**Garden soil, Italy**	**MN633081**	**MT874996**
*K*. *submersum* (*C submersum*)	CBS 101575^T^	River sediments, Spain	NR157445	NG064180
*K*. *terreum*	CBS 342.64^T^	Lawn soil, India	KT155876	KC989709
*K*. *turgidum*	CBS 142596^T^	Barber shop soil, India	KY290503	KY962732
***K*.** ***wagneri***	**BiMM F77**^T^	**Forest soil, Slovakia**	**MN633083**	**MT874999**
*Ct*. *serratus*	CBS 187.61^T^	Soil, Australia	NR144890	AY176733

^a^BiMM, Bioactive Microbial Metabolites Unit, UFT-Tulln, Austria; UAMH, University of Alberta Microfungus Collection and Herbarium; IMI, CAB International Biosciences, Egham, UK; FMR, Facultat de Medicina in Ciències de la Salut, Reus, Spain; CBS (Westerdijk Fungal Biodiversity Institute), Utrecht, The Netherlands; NBRC, NITE Biological Resource Centre, Japan; IFO, Institute for Fermentation, Osaka, Japan; G, EM, and GZUIFR strains, The Institute of Fungus Resource, Guizhou University, China; *A*, *Aphanoascus*; *K*, *Keratinophyton*; *C*, *Chrysosporium*; *Ct*, *Ctenomyces*; ^T^, ex-type culture. Data in bold generated in the present study

### Phylogenetic analysis

For phylogenetic analysis, sequences were aligned with ClustalX (Larkin et al. 2007). Phylogenetic analysis based on ITS locus was performed using GTR + I + G4 + F model with 1000 bootstrap replicates on IQ-TREE web server (Trifinopoulos et al. 2016) and ITS-LSU combined data phylogeny was constructed using MRBAYES v3.2.7adev (Ronquist and Huelsenbeck 2003) with default settings on the CIPRES portal (http://www.phylo.org/). *Ctenomyces serratus* (type species CBS 187.61) was used as an outgroup. TREEVIEW v1.6.6 (Page 1996) and iTOL v6 (Letunic and Bork 2019) were used to display and edit phylogenetic trees.

## RESULTS

### Morphological analyses and keratin degradation

The results of the morphological analyses are given for each novel species under the Taxonomy section below. Temperature dependent growth of the new *Keratinophyton* species on PDA, MEA and SDA after 14 d are provided in Table S1a–c. Briefly, *K*. *lemmensii* grew better than the other three new species on the same type of media and at the same incubation temperatures. All species showed good growth at 20–25 °C on all three media.

Ability to digest keratin after 21 d was observed in all four new species on both testing media (PDA and YEW). However, a value of attack intensity on the hair according to the scale of Marchisio et al. (1994) differed substantially amongst the species. It was very strong in *K. gollerae* and *K. straussii* (=4), moderate in *K. wagneri* (=2), and weak in *K. lemmensii* (= 0–-1) (Fig. 10).

### Phylogenetical analysis

**Fig. 1:**
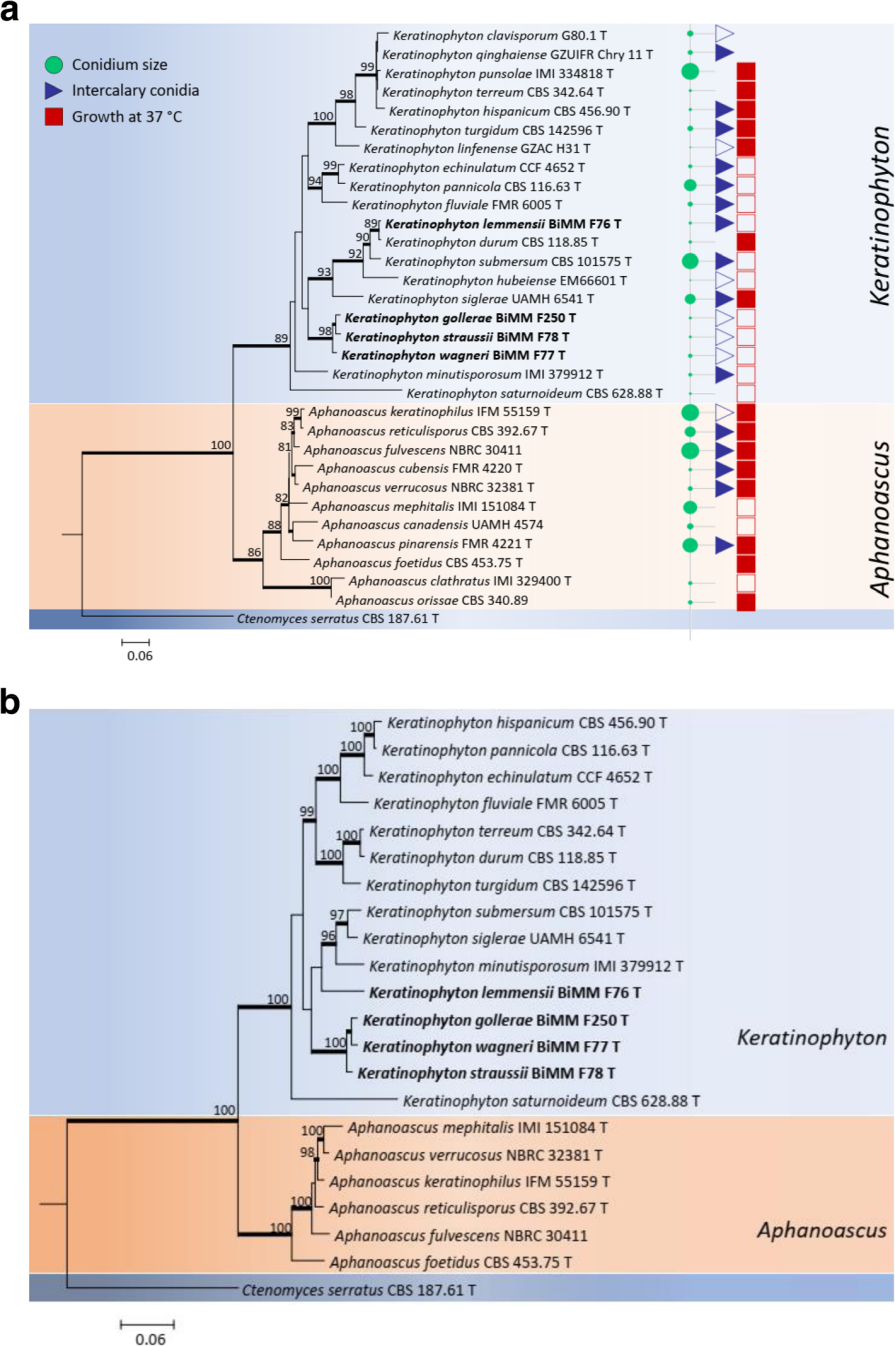
**a** Maximum Likelihood (ML) tree based on ITS sequence for the new taxa of *Keratinophyton* is compared with available sequences of the other related species as well as their conidium size, presence of intercalary conidia and ability to grow at 37 °C. Numbers at nodes indicate bootstrap values. *Ctenomyces serratus* was used as outgroup. A sequence for *K*. *multiporum* was not available for the study. T, ex-type culture. New species are shown in bold. **b** Bayesian interference tree based on combination of ITS and LSU rDNA sequences for new taxa of *Keratinophyton* together with available sequences of the other related species. Numbers at nodes indicate bootstrap values. *Ctenomyces serratus* was used as outgroup. New species are shown in bold

The phylogenetic tree of ITS dataset (*n* = 32) was 551 bp in length which had 286 variable and 200 parsimony-informative sites. ITS phylogeny indicated the presence of six terminal clusters in the monophyletic *Keratinophyton* clade with high bootstrap support and low interspecific sequence divergence (Fig. 1a). *Keratinophyton saturnoideum* and *K*. *minutisporosum* formed a basal branch to the clade. Isolate BiMM-F76 (*K. lemmensii* sp. nov.) was close to *K*. *durum* (with 99% ITS and 95% LSU similarity) and clustered also with *K. hubeiense* and *K. submersum*. In addition, *K. straussii* sp. nov., *K. gollerae* sp. nov., and *K. wagneri* sp. nov., represented by the ex-type cultures BiMM-F78, BiMM-F250 and BiMM-F77, respectively, were resolved in a separate terminal cluster-lineage. A concatenated phylogeny of ITS and LSU sequences (*n* = 22) was 1094 bp length and included 354 variable and 224 parsimony-informative sites. According to a combined data set analysis, four clusters were found in the *Keratinophyton* clade with *K*. *saturnoideum* as a basal branch (Fig. 1b). Differently from the ITS phylogeny, *K*. *durum* was placed in a different cluster from *K*. *submersum* and *K*. *lemmensii* in the concatenated loci phylogeny (Fig. 1b).

## TAXONOMY

The phylogenetic analyses strongly supported the recent distinct classification of the species previously classified as *Chrysosporium* and only known from asexual morphs into two phylogenetically different genera, *Aphanoascus* and *Keratinophyton* (Sharma and Shouche 2017; Sutton et al. 2013). Species described in *Chrysosporium* which were resolved in a monophyletic clade with *Keratinophyton* are therefore combined into *Keratinophyton* in the present paper and provided together with four new *Keratinophyton* species. The main distinguishing phenotypic characteristics of the four new species were compared with those in the other members of the genus that are also unable to produce ascomata (Table 2).

**Table 2:** Comparison of the key phenotypic characteristics of *Keratinophyton* species

Species	Growth at 30 °C on PDA^a^	Colony color, growth/ reverse on PDA at 25 °C, after 14d^b^	Conidial shape	Conidial dimensions (μm)	Conidial surface	Intercalary conidia	References
*K. gollerae* **sp. nov.**	None	White to creamy, 20–22 mm/white to yellowish	Obovoid to clavate	5.0–7.0 **×** 2.0–2.5	Smooth to finely roughened	Absent	**This study**
*K. lemmensii* **sp. nov.**	Present (good)	White, 28–35 mm/lemon yellow	Clavate to filiform	3.0–40 μm (1- to 2-celled)	Smooth	Present	**This study**
*K. straussii* **sp. nov.**	Present (good)	White to creamy, 24–28 mm/white to yellowish	Obovoid to clavate	4.5–5.0 × 2.5–3.0	Verrucose	Absent	**This study**
*K. wagneri* **sp. nov.**	Present (restricted)	White to yellowish, 25–30 mm/white to yellowish	Obovoid to clavate	4.0–8.0 × 2.5–4.0	Verrucose	Absent	**This study**
*K. clavisporum*	Present (restricted)^e^	White, 53 mm (26 °C)/red-brown	Clavate to long -ellipsoidal	5.0–10 × 2.5–5.0	Smooth	Absent	Zhang et al. 2017
*K. echinulatum*	Present (good)	Yellow to pale orange yellow, 28–45 mm/orange yellow	Obovoid to clavate	4.5–7.0 × 2.5–4.0	Echinulate	Present	Hubka et al. 2016
*K. fluviale*	Present (good)	White to yellowish white, 60–70 mm (30 °C)/brownish orange	Obovate, clavate, nearly ellipsoidal or pyriform	3.5–15 × 2.0–3.0 (1-and 2-celled)	Verrucose	Present (very rare)	Vidal et al. 2000
*K. qinghaiense*	Present (good)^d^	White to yellowish, 30 mm (7 days)/yellowish	Clavate to cylindrical	3.6–13 × 1.8–3.6	Smooth	Present	Han et al. 2013
*K. hubeiense*	Present (restricted)^e^	Grey white to white, 65–67 mm/reverse yellowish	Obovoid to ellipsoidal	2.2–4.3 × 1.6–3.2	Smooth	Absent	Zhang et al. 2016
*K. linfenense*	Present (good)	White to cream, 72 mm (30 °C)/white to light yellow	Ellipsoidal to fusiform, also clavate	3.2–5.4 x-1.4–2.2	Smooth	Absent	Liang et al. 2009
*K. minutisporosum*	Present (good)	White to yellowish white, 55–70 mm/white	Pyriform or subglobose, also clavate	3.0–4.0 (−11) × 1.5–3.5	Verrucose	Present (very rare)	Vidal et al. 2002
*K. pannicola*	Present (good)	White to pale yellow, 20–38 mm /pale brown^c^	Obovoid to clavate	6.0–11 × 3.5–4.5	Verrucose	Present (less abundant)	van Oorschot 1980
*K. siglerae*	Present (good)	Griseous orange, 15–20 mm (21 d)/ pale brown	Cylindrical to clavate	5.0–30 × 2.0–3.5 1-and 2-celled	Smooth to slightly verrucose	Present	Cano and Guarro 1994
*K. submersum*	Present (restricted)	Yellowish white, 50–60 mm/yellowish white	Clavate, also pyriform, obovoid and subglobose	4.0–35 × 2.5–5.0 (1- to 4-celled)	Smooth to verrucose-thick-walled	Present (in old cultures)	Vidal et al. 2002
*K. turgidum*	Present (good)	White, 50–55 mm (SGA at 28 °C)/pale brown	Pyriform to oval	5.0–7.0 × 3.5–5.0	Smooth	Present	Sharma and Shouche 2017

^a^if not stated other medium

^b^if not stated otherwise

^c^PYE, Phytone yeast extract agar

^d^Yanfeng Han personal communication

***Keratinophyton clavisporum*** (Zhang, Han & Liang) Labuda & Bernreiter, **comb. nov.**

MycoBank: MB833653

***Basionym*:** ***Chrysosporium clavisporum*** Y.W. Zhang, Y.F. Han & Z.Q. Liang - *Phytotaxa* **303**: 177; 2017.

*Type*: GZUIFR-G80.1; isolated from plant root soil by Y. Luo, China. For detailed description of the species, see the Zhang et al. (2017).

***Keratinophyton echinulatum*** (Hubka, Mallátová, Čmoková & Kolařík) Labuda & Bernreiter, **comb. nov.**

MycoBank: MB833636

*Basionym*: *Chrysosporium echinulatum* Hubka, Mallátová, Čmoková & M. Kolařík - *Persoonia* **36**: 410; 2016.

*Type*: CCF 4652 = CBS 141178 = UAMH 11824; from sole of the foot by N. Mallátová, Czechia. For detailed description of the species, see the Hubka et al. (2016).

***Keratinophyton fluviale*** (Vidal & Guarro) Labuda & Bernreiter, **comb. nov.**

MycoBank: MB8333637

*Basionym*: *Chrysosporium fluviale* Vidal & Guarro - *Mycol. Res.* **104**: 245; 2000.

*Type*: CBS 100809 = FMR 6005 = IMI 378764, isolated from river sediments, by P. Vidal, Spain. For detailed description of the species, see the Vidal et al. (2000).

***Keratinophyton gollerae*** Labuda, Bernreiter, Kubátová, Schüller & Strauss, **sp. nov.**

(Figs. 2 and 3)

**Fig. 2:**
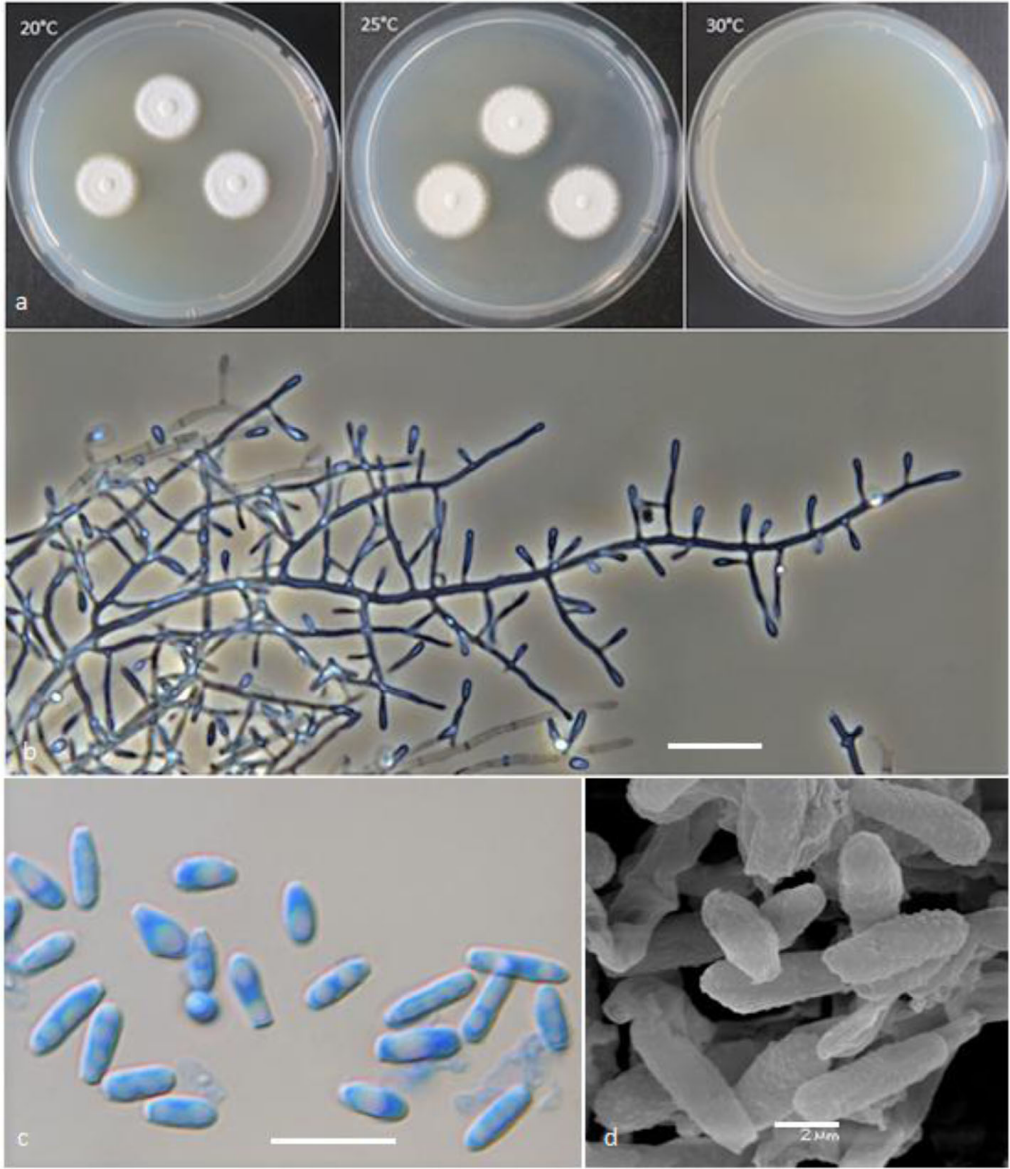
*Keratinophyton gollerae* (BiMM-F250). **a** Colonies on PDA (after 14 d) at 20 °C, 25 °C and 30 °C. **b** Conidiophores with aleurioconidia. **c** Aleurioconidia and arthroconidia (on PDA, after 14 d). **d** Scanning electron microscopy (SEM) of aleurioconidia (on PDA, after 14 d). Bars = 20 μm (**b**), 10 μm (**c**), 2 μm (**d**)

**Fig. 3:**
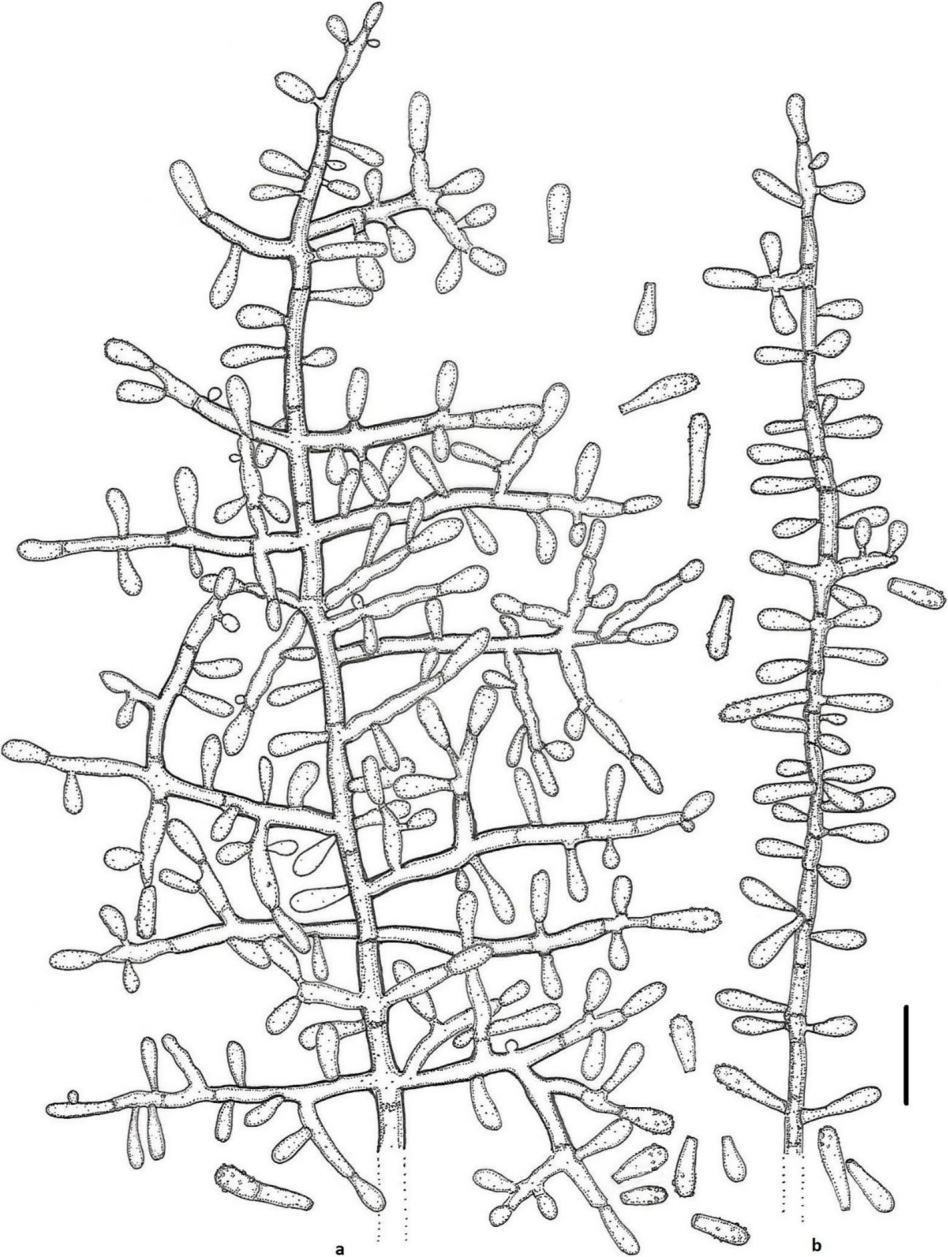
Line drawing of micromorphology of *Keratinophyton gollerae* (BiMM-F250). **a**, **b** Conidiophores with young and mature aleurioconidia on PDA (after 14 d). **a** Branched conidiophore. **b** Unbranched conidiophore with sessile aleurioconidia. Bar = 10 μm

*MycoBank*: MB833633

*Etymology:* Named in honour of Sabine Strauss-Goller, Department of Applied Genetics and Cell Biology, Fungal Genetics and Genomics Laboratory, University of Natural Resources and Life Sciences, Vienna (BOKU), Austria, an expert in the fungal genetics and indoor mould analyses.

*Type*: **Slovak Republic:** Tatranská Lomnica, from forest soil, Jul. 2019, *R. Labuda* (PRM 952499 – holotype; BiMM-F250 = CCF 6360 – ex-type cultures). ITS sequence, GenBank MN633084; LSU sequence, GenBank MT874997.

*Description*: *Sexual morph* not observed on any of the media used. *Asexual morph* on PDA. *Vegetative mycelium* of hyaline, septate, smooth-walled, sparsely to pronouncedly branched hyphae, often at right angles, 1.0–5.0 μm diam. *Racquet hyphae* present. *Conidia* (aleurioconidia), hyaline, white in mass, thin-walled, mostly smooth to finely roughened, some also verrucose (light microscope) and irregularly ornamented with minute warts (SEM). Terminal and lateral conidia born on main fertile hyphae or from side branches of variable length, sessile or on short protrusions, occasionally only very slightly swollen and of variable length, solitary, 1–3 (− 5) per conidiogenous cell, obovate to clavate, mostly 1-celled, (3.5–)5.0–7.0(− 10.0) x (1.5–)2.0–2.5(− 3.0) μm (mean = 5.2 ± 0.9 × 2.2 ± 0.2 μm, *n* = 120). *Intercalary conidia* not observed. *Chlamydospores* not observed.

*Culture characteristics*: *Colonies* on PDA 20–22 mm diam at 25 °C, after 14 d, powdery to downy (mealy), with abundant sporulation, white to creamy, flat, umbonate at the centre, with regular colony margin submersed into agar, reverse white to slightly yellowish, no pigment or exudate produced. At 30 °C, no growth (germination only). *Colonies* on SDA 23–25 mm diam at 25 °C, after 14 d, morphology similar to when on PDA with more floccose colony margin and more yellowish colonies, with dark yellow reverse. At 30 °C, no growth (no germination). *Colonies* on MEA 14–16 mm diam at 25 °C, after 14 d, morphology similar to PDA with more floccose colonies and with yellow reverse. At 30 °C, no growth (no germination). *Colonies* on CMA and PCA attaining 15–20 mm diam at 25 °C, after 21 d, white, granular, with good sporulation, reverse yellowish. No ascomata observed after prolonged incubation (3 months). The *optimum temperature* for growth on PDA, SDA and MEA 15–25 °C (Table S1a–c). *Minimum growth* (microcolonies to 1 mm in diam) at 10 °C. Germination of the conidia observed at 8 °*C. The maximum temperature* for growth on PDA 29 °C, while 27 °C and 28 °C on MEA and SDA, respectively (microcolonies to 1 mm diam). *Keratinolytic activity* very strong (Fig. 10b), with hair attack intensity = 4. *Urease activity* negative (after 14 d of incubation).

*Diagnosis: Keratinophyton gollerae* molecularly can be distinguished from other *Keratinophyton* species by ITS locus analysis. Combination of the following phenotypic features can be used to differentiate this fungus from other species in the genus: (1) obovoid-clavate and smooth to finely roughened conidia, (2) No growth at 30 °C, and (3) yellowish colonies with dark yellow reverse at 25 °C on SDA.

*Notes:* Based on a search of NCBI GenBank nucleotide database, the closest hit for *K. gollerae* using the ITS sequence is *K. minutisporosum* (as *Chrysosporium minutisporosum* CBS 101577; GenBank acc. KT155616), with identity = 487/543 (90%) and gaps 11/543 (2%). Phenotypically, *K. gollerae* can be readily distinguished from the *K*. *minutisporum* by its smooth to finely roughened larger conida (5–7 × 2–2.5 μm vs. 3–4 × 1.5–3.5 μm), dark yellow colony reverse at 25 °C on PDA. Based on ITS phylogeny (Fig. 1a), *K*. *gollerae* formed a cluster together with *K. straussii* and *K. wagneri*, and it can be differentiated by its inability to grow at 30 °C, narrower and mostly smooth to finely roughened conidia, and its slower growth at 25 °C on PDA. Moreover, in comparison with *K. straussii*, *K. gollerae* grows substantially faster at 15 °C (on PDA and SDA) and its conidia germinate at 8 °C (see Table S1a–c).

***Keratinophyton hubeiense*** (Zhang, Han & Liang) Labuda & Bernreiter, **comb. nov.**

MycoBank: MB833638

*Basionym*: *Chrysosporium hubeiense* Yan W. Zhang, Y.F. Han & Z.Q. Liang - *Phytotaxa* **270**: 213; 2016.

*Type*: GZAC EM66601, isolated from soil under the chicken feather by Y.R. Wang, China. For detailed description of the species, see the Zhang et al. (2016).

***Keratinophyton lemmensii*** Labuda, Bernreiter, Kubátová & Schüller, **sp. nov.**

(Figs. 4 and 5)

**Fig. 4:**
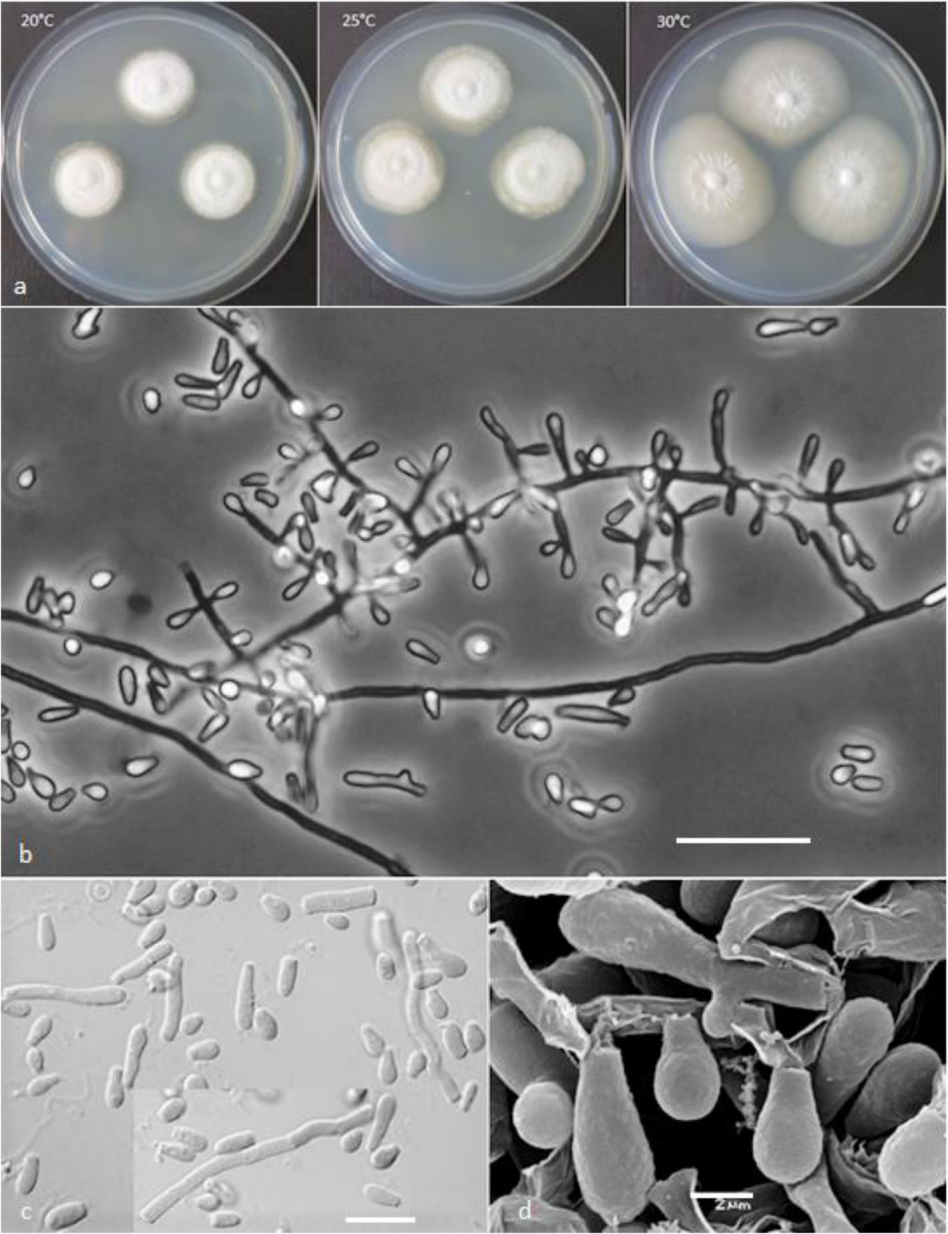
*Keratinophyton lemmensii* (BiMM-F76). **a** Colonies on PDA (after 14 d) at 20 °C, 25 °C and 30 °C. **b** Conidiophores with aleurioconidia. **c** Aleurioconidia and arthroconidia (on PDA, after 14 d). **d** Scanning electron microscopy (SEM) of aleurioconidia (on PDA, after 14 d). Bars = 20 μm (**b**), 10 μm (**c**), 2 μm (**d**)

**Fig. 5:**
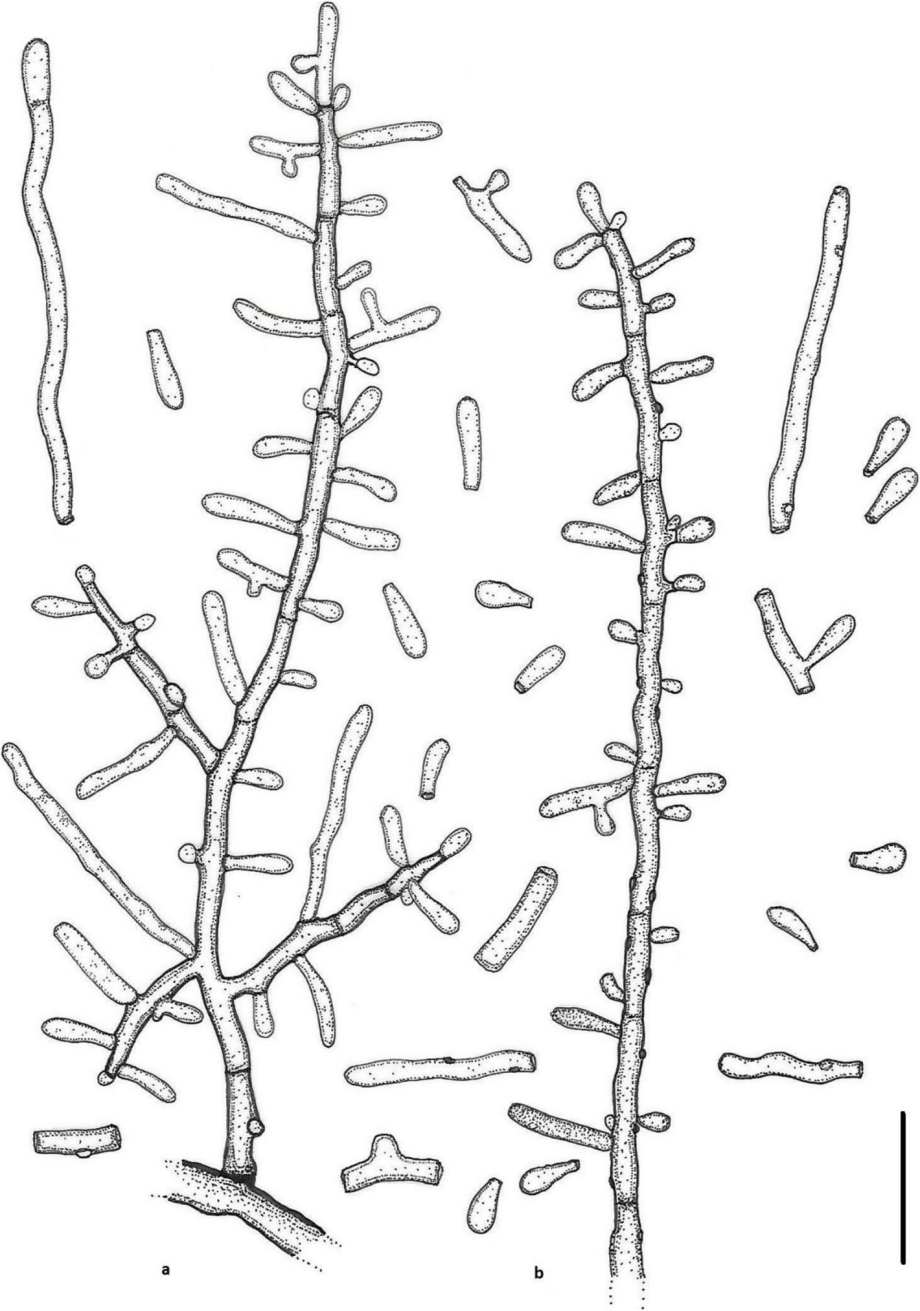
Line drawing of micromorphology of *Keratinophyton lemmensii* (BiMM-F76). **a**, **b** Conidiophores with young and mature aleurioconidia, including arthroconidia on PDA (after 14 d). **a** Branched conidiophore. **b** Unbranched conidiophore with sessile aleurioconidia. Bar = 10 μm

*MycoBank*: MB833632

*Etymology*: Named in honour of Marc Lemmens, Department of Plant Protection, University of Natural Resources and Life Sciences, Vienna (BOKU), Austria, an expert in fungal plant pathology.

*Type*: **Austria:** Tulln and der Donau, from compost soil at IFA Tulln, Aug. 2015, *R. Labuda* (PRM 952498 –holotype; BiMM-F76 = CCF 6359 – ex-type cultures). ITS sequence GenBank MN633082; LSU sequence GenBank MT874998.

*Description: Sexual morph* not observed on any of the media used in the present study. *Vegetative mycelium* consisting of hyaline, smooth-walled, septate, sparsely branched hyphae, 1.5–5.0 μm diam. *Racquet hyphae* present. *Conidia* aleuroconidia, hyaline, white in mass, thin-walled, smooth to sparsely irregularly ornamented with minute warts (SEM); terminal and lateral conidia born on main fertile hyphae as sessile or on short protrusions, solitary, 1–3 (− 5) per *conidiogenous cell*, obovate to clavate, 1-celled, (3.0–)4.5–6.5(− 7.5) x (1.5–)2.0–2.5(− 4.0) μm (mean = 4.9 ± 0.8 × 2.4 ± 0.4 μm, *n* = 120), and filiform, often sinusoidal, 1- to 2-celled, 25–35(− 40) μm long conidia also present. *Intercalary conidia* (arthroconidia) present, 10–15 μm long. *Chlamydospores* not observed.

*Culture characteristics*: *Colonies* on PDA 28–35 mm diam at 25 °C, after 14 d, floccose, with good sporulation, white, flat, slightly elevated (umbonate) at the centre, with irregular margin, reverse lemon yellow, soluble pigment bright yellow, a few small clear to yellow-orange exudate droplets produced. At 30 °C, 38–45 mm diam after 14 d, white, flat, floccose and radially sulcate with good sporulation only at the centre, and with lemon yellow reverse. *Colonies* on SDA 28–35 mm diam at 25 °C, after 14 d, morphology similar to PDA, without exudate and with pale yellow reverse. At 30 °C, 30–32 mm diam after 14 d, white, flat, floccose with good sporulation, with pale yellow reverse. *Colonies* on MEA 20–25 mm diam at 25 °C after 14 d, morphology similar to PDA, exudate absent, and pale-yellow reverse. At 30 °C, 18–20 mm diam after 14 d, white, floccose and radially sulcate, with good sporulation, and with pale yellow reverse. *Colonies* on CMA and PCA, 45–50 mm diam at 25 °C, after 21 d, white, flat and spread with poor sporulation, reverse white. No ascomata observed after prolonged incubation (3 months). *The optimum temperature* on PDA, SDA and MEA 25–30 °C (Table S1a–c). *Minimum growth* (1–2 mm diam) at 8 °C. *The maximum temperature* for growth 32 °C (microcolonies to 1 mm diam). *Keratinolytic activity* very weak (Fig. 10a), with hair attack intensity = 0–1. *Urease activity* positive (after 3 d of incubation).

*Diagnosis*: This species molecularly can be distinguished from other *Keratinophyton* species by ITS locus analysis. Phenotypically, *K. lemmensii* is unique and differs from the relatives in the same clade based on ITS phylogeny (*K. durum*, *K. hubeiense*, *K. submersum*, and *K. siglerae*) by the combination of the following features: (1) presence of long filiform often sinusoidal uni- to bicellular conidia (to 40 μm), (2) white, moderately fast growing colonies (28–35 mm diam, on PDA at 25 °C), (3) production of lemon yellow pigment on PDA at 25 °C, (4) minimum 8 °C and maximum 32 °C growth temperature, (5) very weak keratin digestion after 21 d. Presence of filiform often sinusoidal conidia and abundant arthroconidia, production of bright yellow pigment on PDA and good growth at 30 °C.

*Notes:* Based on a search of NCBI GenBank nucleotide database, the closest hit for *K. lemmensii* using the ITS sequence was *K. durum* (FMR5651; GenBank acc. AJ439434; identities = 568/577 (98%), gaps 0/577 (0%). However, *K. lemmensii* can be directly distinguished from *K. durum* by its asexual morph also by the presence of numerous arthroconidia which are completely missing in the latter species (Cano and Guarro 1990; Currah 1985).

***Keratinophyton linfenense*** (Liang, Liang & Han) Labuda & Bernreiter, **comb. nov.**

MycoBank: MB833639

*Basionym*: *Chrysosporium linfenense* Z.Q. Liang, J.D. Liang & Y.F. Han - *Mycotaxon* **110**: 67; 2009.

*Type*: GZUXIFR H31, isolated from rhizosphere soil by G. Don, China. For detailed description of the species, see Liang et al. (2009).

***Keratinophyton minutisporosum*** (Vidal & Guarro) Labuda & Bernreiter, **comb. nov.**

MycoBank: MB833640

*Basionym*: *Chrysosporium minutisporosum* P. Vidal & Guarro - *Stud. Mycol.* **47**: 205; 2002.

*Type*: CBS 101577 = IMI 379912 = FMR 6096 isolated from river mouth sediment by P. Vidal, Spain. For detailed description of the species, see Vidal et al. (2002).

***Keratinophyton pannicola*** (Corda) Labuda & Bernreiter, **comb. nov.**

MycoBank: MB8333643

*Basionym*: *Capillaria pannicola* Corda - *Icon. Fung*.**1**: 10; 1837.

≡ *Sporotrichum pannicola* (Corda) Rabenh. - *Deutschl. Krypt.-Fl.* **1**: 78; 1844.

≡ *Chrysosporium pannicola* (Corda) Oorschot & Stalpers - *Stud. Mycol.* **20**: 43; 1980.

*Synonym*: *Trichophyton evolceanui* H.S. Randhawa & R.S. Sandhu - *Mycopath. Mycol. Appl.* **20**: 232; 1963.

≡ *Chrysosporium evolceanui* (Randhawa & Sandhu) Garg - *Sabouraudia* **4**: 262; 1966.

*Type*: CBS 116.63 = ATCC 22400 = IHEM 4436 = IMI 147545 = NCPF 489 = RV 26475 = UAMH 1275, isolated from soil by Randhawa & Sandhu, India.

***Keratinophyton siglerae*** (Cano & Guarro) Labuda & Bernreiter, **comb. nov.**

MycoBank: MB833641

*Basionym*: *Chrysosporium siglerae* Cano & Guarro - *Mycotaxon* **51**: 75; 1994.

*Type*: UAMH 6541 = FMR 3066 = IMI 336467, isolated from garden soil, Spain. For detailed description of the species, see Cano and Guarro (1994).

***Keratinophyton submersum*** (Vidal & Guarro) Labuda & Bernreiter, **comb. nov.**

MycoBank: MB833642

*Basionym*: *Chrysosporium submersum* P. Vidal & Guarro - *Stud. Mycol.* **47**: 200; 2002.

Type: CBS 101575 = IMI 379911 = FMR 6088, isolated from river mouth sediment by P. Vidal, Spain. For detailed description of the species, see Vidal et al. (2002).

***Keratinophyton straussii*** Labuda, Bernreiter, Kubátová & Schüller, **sp. nov.**

(Figs. 6 and 7)

**Fig. 6:**
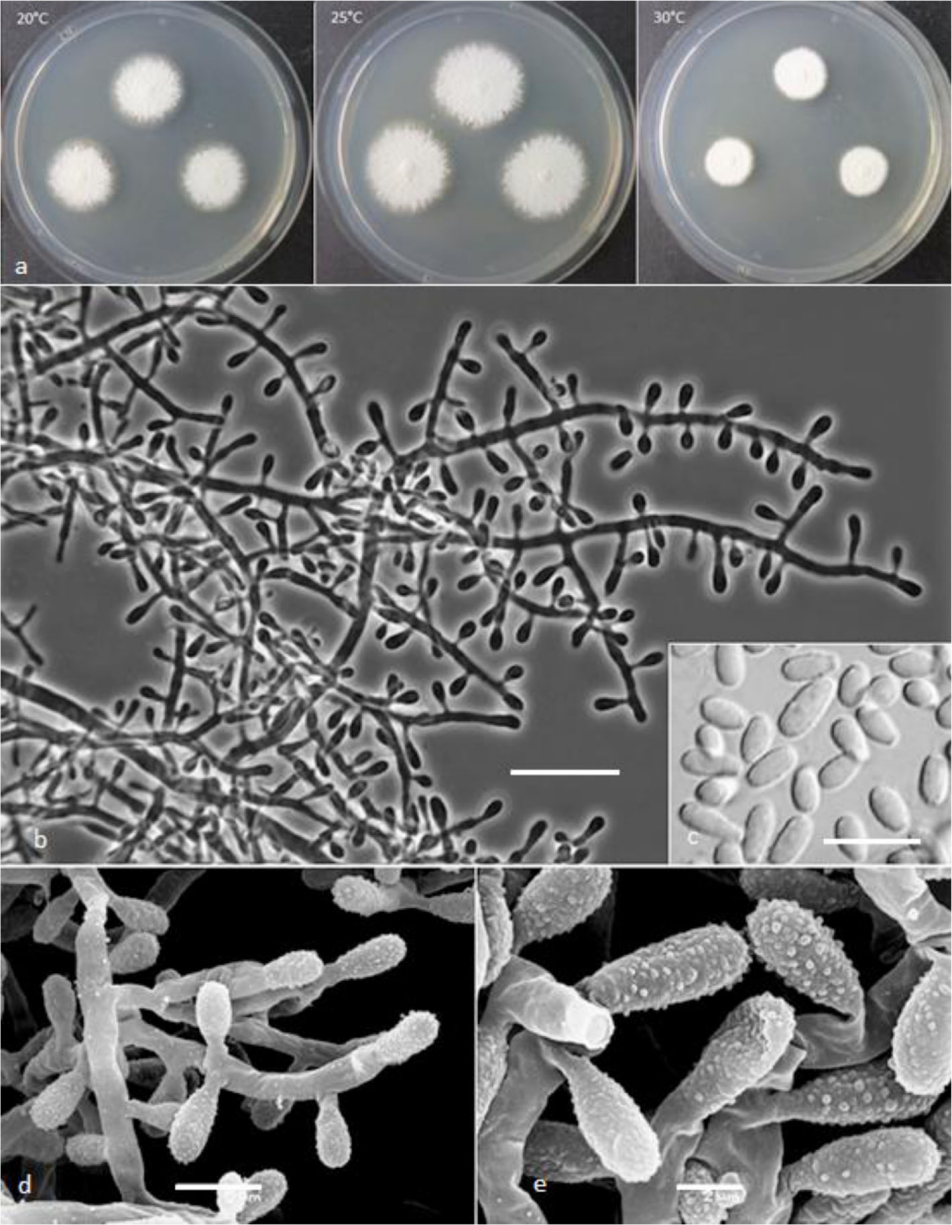
*Keratinophyton straussii* BiMM-F78. **a** Colonies on PDA (14 d old) at 20 °C, 25 °C and 30 °C. **b** Conidiophores with aleurioconidia. **c** Aleurioconidia (on PDA, after 14 d). **d**, **e** Scanning electron microscopy (SEM) of conidiogenous cells and aleurioconidia (on PDA, after 14 d). Bars = 20 μm (**b**), 10 μm (**c**), 5 μm (**d**), 2 μm (**e**)

**Fig. 7:**
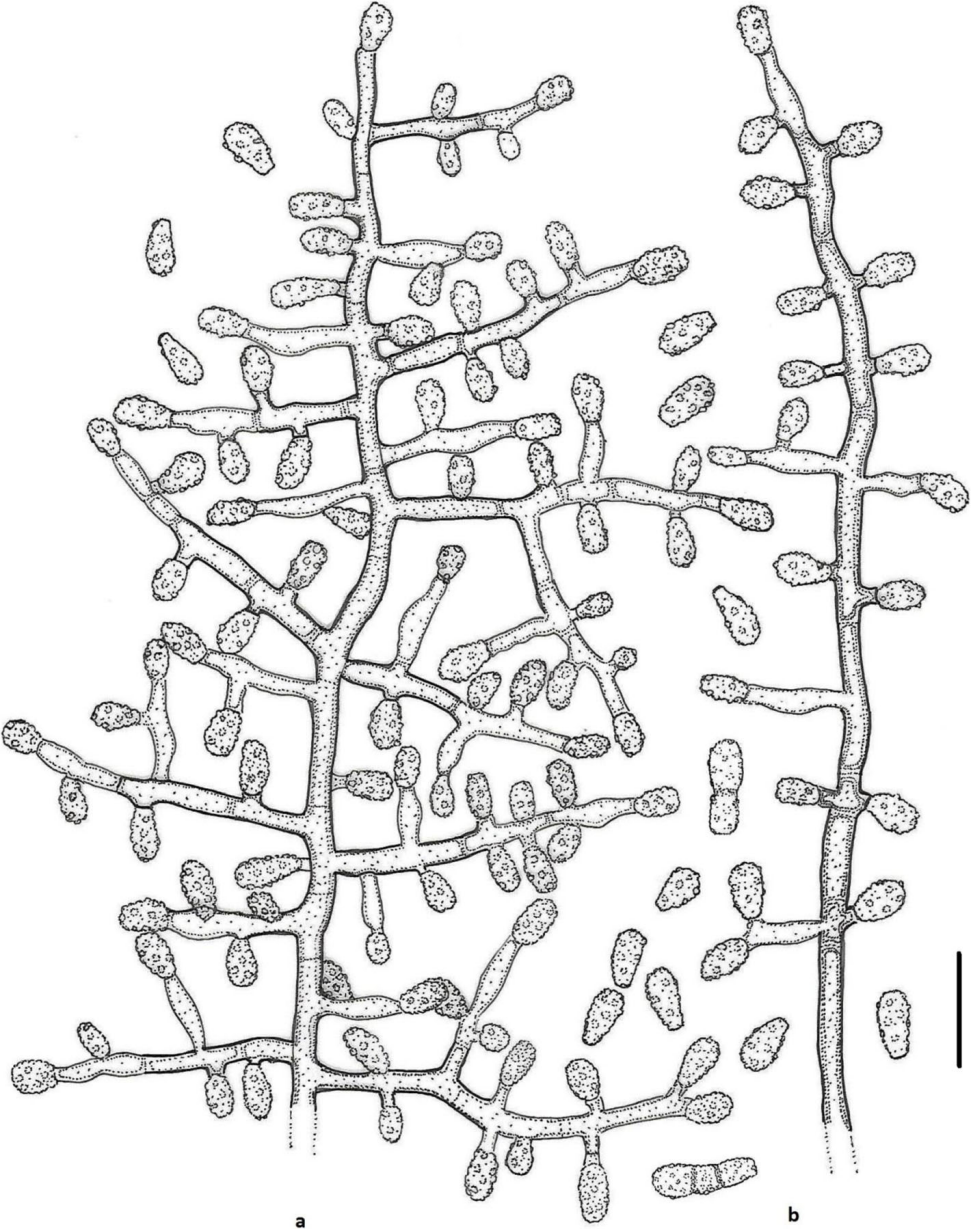
Line drawing of micromorphology of *Keratinophyton straussii* (BiMM-F78). **a**, **b** Conidiophores with young and mature aleurioconidia on PDA (14 d old). **a** Branched conidiophore. **b** Unbranched conidiophore with sessile aleurioconidia. Bar = 10 μm

*MycoBank*: MB833634

*Etymology*: Named in honour of Joseph Strauss, Head of the Department of Applied Genetics and Cell Biology, founder of the Fungal Genetics and Genomics Laboratory, University of Natural Resources and Life Sciences, Vienna (BOKU), Austria, and an expert in fungal genetics, epigenetics and functional genomics.

*Type*: **Italy**: Vieste, from garden soil, Aug. 2015, *R. Labuda* (PRM 952500 – holotype; BiMM-F78 = CCF 6361 – ex-type cultures). ITS sequence, GenBank MN633081; LSU sequences, GenBank MT874996.

*Description*: *Sexual morph* not observed on any of the media used. *Asexual morph* on PDA. *Vegetative mycelium* of hyaline, septate, smooth-walled, sparsely to pronouncedly branched hyphae, usually at right angles, 1.5–4.0 μm diam. *Racquet hyphae* present. *Conidia* (aleurioconidia), hyaline, white to yellowish in mass, thin-walled and regularly ornamented with minute warts (SEM) and coarsely roughened (light microscope). Terminal and lateral conidia born on main fertile hyphae or from side branches of variable length, sessile or on short protrusions, commonly slightly swollen, length variable, solitary, 1–3 (5) per conidiogenous cell, obovate to clavate, 1-celled, (3.5–)4.5–5.0(− 6.5) x (2.0–)2.5–3.0(− 3.5) μm (mean = 4.9 ± 0.4 × 2.6 ± 0.2 μm, *n* = 120), very rarely 2- to 3-celled, to 12 μm large aleurioconida also present. *Intercalary conidia* not observed. *Chlamydospores* not observed.

*Culture characteristics*: *Colonies* on PDA 24–28 mm diam at 25 °C, after 14 d, powdery to downy (mealy), with abundant sporulation, white to very slightly creamy yellowish, flat, slightly elevated (umbonate) remaining powdery at the centre, with irregular margin, reverse white with slightly yellowish centre, no pigment or exudate produced. At 30 °C, 15–20 mm diam after 14 d, white to creamy yellowish, flat, powdery to downy (mealy) with very good sporulation, and with white to yellowish reverse. *Colonies* on SDA 16–20 mm diam at 25 °C, after 14 d, morphology as on PDA with dark yellow reverse. In age (after 5 wk) yellow pigment produced and colony reverse becoming bright reddish yellow to orange. At 30 °C, 15–20 mm diam after 14 d, white to creamy yellowish, umbonate, with strong sporulation, and with yellowish reverse. *Colonies* on MEA 18–20 mm diam at 25 °C, after 14 d, morphology as on PDA with more floccose and yellowish. At 30 °C, 5–10 mm diam after 14 d, slightly umbonate, floccose to granular, with very good sporulation white to yellowish, and with yellow reverse. *Colonies* on CMA and PCA 18–20 mm diam at 25 °C, after 21 d, white, granular, good sporulation, reverse yellowish. No ascomata observed after prolonged incubation (3 months). The *optimum temperature* for growth on PDA, SDA and MEA 20–25 °C (Table S1a–c). *Minimum growth* (microcolonies to 1–2 mm diam) at 10 °C. No germination of the spores at 8 °*C. The maximum temperature* for growth 32 °C (microcolonies to 1–2 mm diam). *Keratinolytic activity* very strong (Fig. 10c), with hair degradation intensity = 4. *Urease activity* negative (after 14 d of incubation).

*Diagnosis*: *Keratinophyton straussii* molecularly can be distinguished from other *Keratinophyton* species by ITS locus analysis. Phenotypically, it can be differentiated by combination of the ability to grow at 30 °C, white to creamy colonies with white to yellowish revers at 25 °C on PDA and conidia morphology (obovoid to clavate and verrucose) (Table 2).

*Additional material examined*: **Italy**: Vieste, from garden soil, isolated from different sub-samples, July 2019, *R*. *Labuda* RL-05 ITS sequence, MT898644; LSU sequence, MT898648); ibid., RL-06 (ITS sequence, MT898645; LSU sequence, MT898649).

*Notes*: Based on a search of the NCBI GenBank nucleotide database, the closest hit for *K. straussii* using the ITS sequence was *K. minutisporosum* (as *Chrysosporium minutisporosum* CBS 101577; GenBank acc. KT155616), with identity = 489/543 (90%) and gaps 10/543 (1%). Two species can be differentiated from each other based on growth rate and colony reverse at 25 °C on PDA (Table 2). Additionally, *K*. *straussii* differs from *K*. *wagnerii* by its ability to grow at 30 °C and strong keratinolytic activity. For the morphological differences between *K*. *gollerae* and *K*. *straussii*, see under *K*. *gollerae*. Additional strains RL-05 and RL-06 grew relatively better (to 5 mm larger diam) than the ex-type culture at 30 °C.

***Keratinophyton qinghaiense*** (Han, Liang & Liang) Labuda & Bernreiter, **comb. nov.**

MycoBank: MB833655

***Basionym*:** ***Chrysosporium*** *qinghaiense* Y.F. Han, J.D. Liang & Z.Q. Liang - *Mycosystema* **32**: 607, 2013.

Type: GZAC GZUIFR-Chry 11, from farmland soil by, Y.F. Han, China.

***Keratinophyton wagneri*** Labuda, Bernreiter, Kubátová & Schüller, **sp. nov.**

(Figs. 8 and 9)

**Fig. 8:**
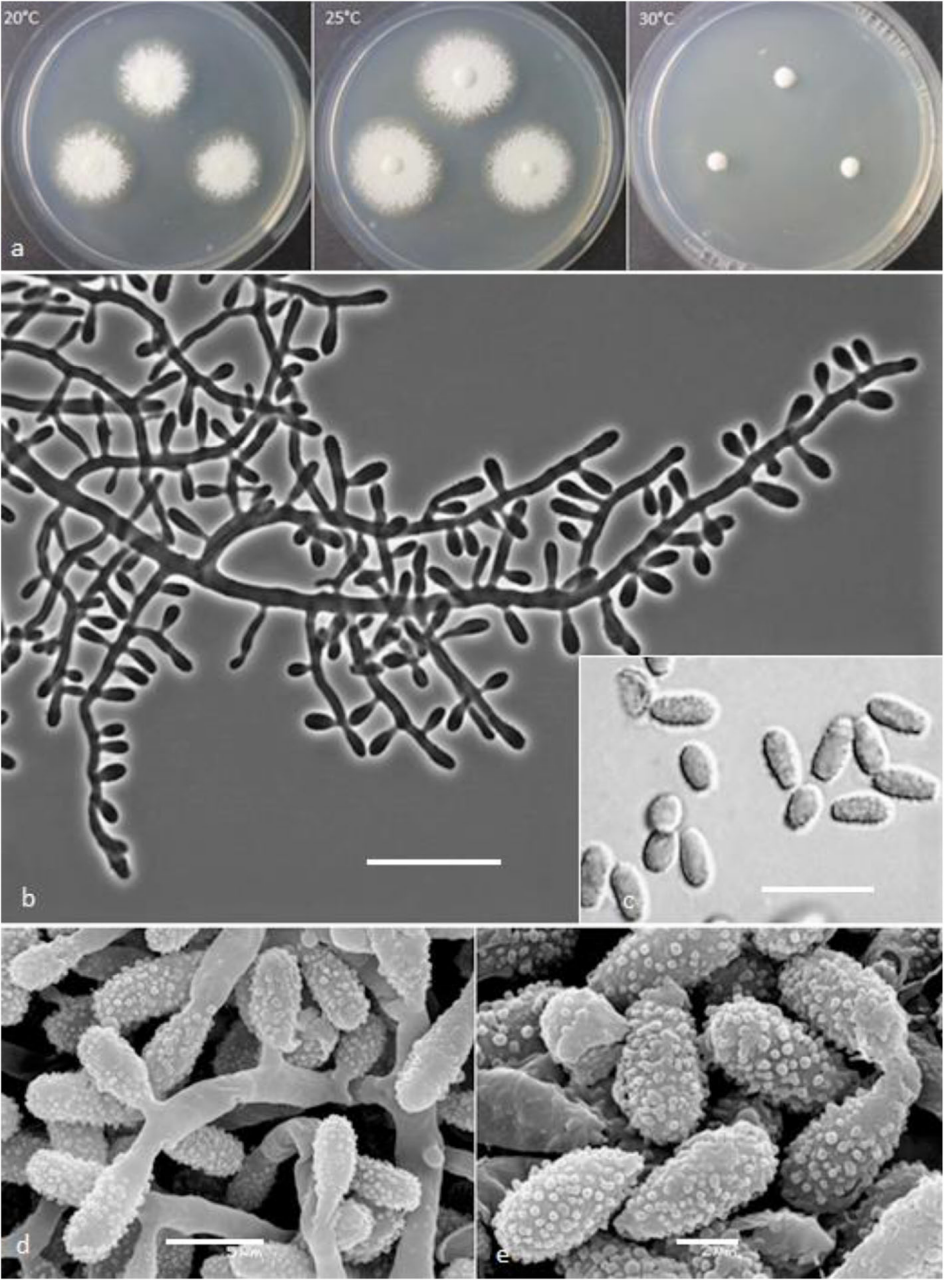
*Keratinophyton wagneri* (BiMM-F77). **a** Colonies on PDA (after 14 d) at 20 °C, 25 °C and 30 °C. **b** Conidiophores with aleurioconidia. **c** Aleurioconidia (on PDA, after 14 d). **d**, **e** Scanning electron microscopy (SEM) of conidiogenous cells and aleurioconidia (on PDA, after 14 d). Bars = 20 μm (**b**), 10 μm (**c**), 5 μm (**d**), 2 μm (**e**)

**Fig. 9:**
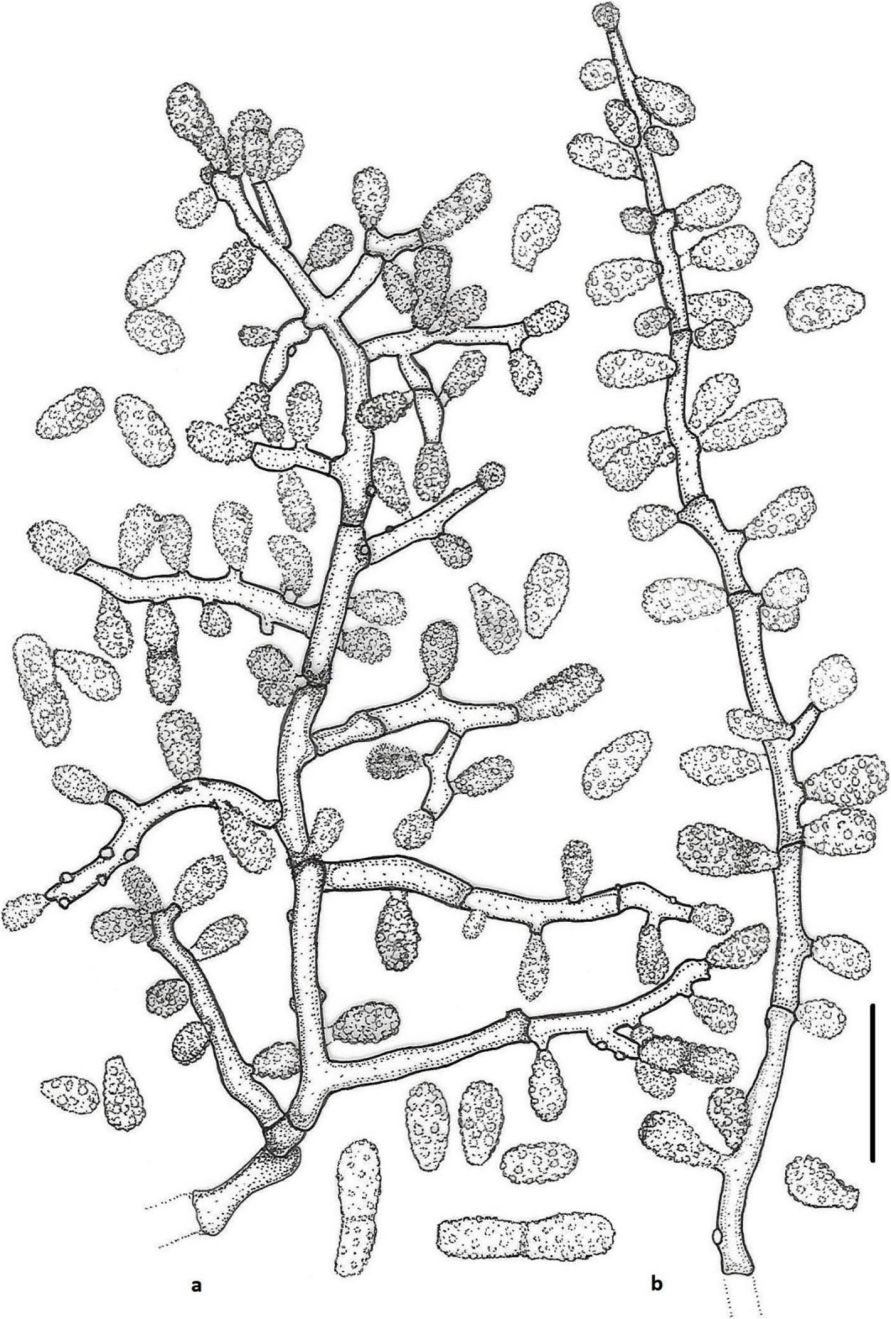
Line drawing of micromorphology of *Keratinophyton wagneri* (BiMM-F77). **a**, **b** Conidiophores with young and mature aleurioconidia on PDA (after 14 d). **a** Branched conidiophore. **b** Unbranched conidiophore with sessile aleurioconidia. Bar = 10 μm

*MycoBank*: MB 833635.

*Etymology*: Named in honour of Martin Wagner, Head of the Unit for Food Microbiology and Head of Institute for Food Safety, Food Technology and Veterinary Public Health, University of Veterinary Medicine, Vienna (Austria), an expert in veterinary microbiology.

*Type*: **Slovak Republic:** Tatranská Lomnica, from forest soil, Aug. 2015, *R. Labuda* (PRM 952501 – holotype; BiMM-F77 = CCF 6362 – ex-type cultures). ITS sequence, GenBank MN633083; LSU sequence, GenBank MT874999.

*Description*: *Sexual morph* not observed on any of the media used. *Asexual morph* on PDA. Vegetative mycelium hyaline, septate, smooth-walled, sparsely to pronouncedly branched hyphae, 2.0–6.0 μm diam. *Racquet hyphae* present. *Conidia* (aleurioconidia), hyaline, white to yellowish in mass, thin-walled and regularly ornamented with minute warts (SEM) and coarsely roughened (light microscope). Terminal and lateral conidia born on main fertile hyphae or from side branches of variable length, sessile or on short protrusions, occasionally swollen and of variable length, solitary, 1–4 (− 10) per conidiogenous cell, obovate to clavate, single celled, (4.0–) 5.5–6.5 (− 8.0) x (2.5–) 3.0–3.5(− 4.0) μm (mean = 5.7 ± 0.4 × 3.2 ± 0.2 μm, *n* = 120), rarely 2-celled, up to 12 μm large ones also present. *Intercalary conidia* not observed. *Chlamydospores* not observed.

**Fig. 10:**
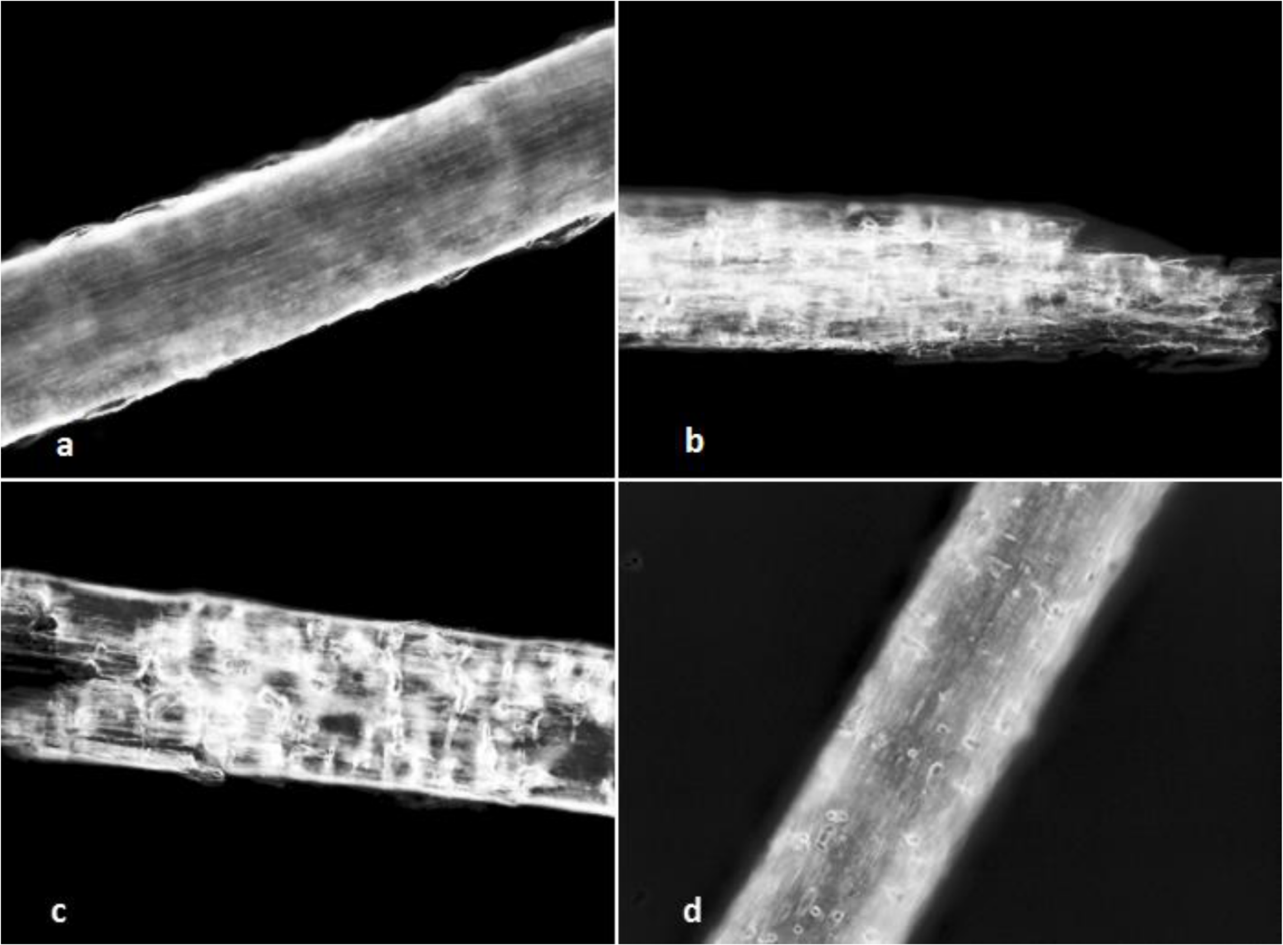
Hair perforation in vitro – keratinolysis; detail of a child’s hair after colonization by the fungus on PDA (after 21 d) at 25 °C. **a** *Keratinophyton lemmensii* (BiMM-F76). **b** *Keratinophyton gollerae* (BiMM-F250). **c** *Keratinophyton straussii* (BiMM-F78). **d** *Keratinophyton wagneri* (BiMM-F77). Intensity of attack on the hair estimated on a scale of 0 to 4 (Marchisio et al. 1994). **a** 0–1 = light attack to cuticle. **b**, **c** 4 = cuticle and cortex attack with about 80% destruction. **d** 2 = cuticle and cortex attack with about 20% destruction

*Culture characteristics*: *Colonies* on PDA 25–30 mm diam at 25 °C, after 14 d, powdery to downy (mealy), with abundant sporulation, white to slightly yellowish, flat, slightly elevated (umbonate) and more floccose at the centre, margin irregular, reverse white with slightly yellowish centre, no pigment or exudate produced. At 30 °C, 4–8 mm diam after 14 d, white, floccose with poor sporulation, and with yellowish reverse. *Colonies* on SDA 14–18 mm diam at 25 °C, after 14 d, morphology similar to PDA. In age, yellowish brown (amber) pigment produced and colony reverse becoming dark reddish brown (after 4 wk). At 30 °C, no growth or only microcolonies. *Colonies* on MEA 18–22 mm diam at 25 °C, after 14 d, morphology as on PDA but more yellowish. At 30 °C, no growth or only micro-colonies produced. *Colonies* on CMA and PCA 20–25 mm diam at 25 °C, after 21 d, white to yellowish, granular, good sporulation, reverse yellowish. Pinkish pigment after 3–4 wk. on PCA (in both tested strains). No ascomata observed after prolonged incubation (3 months). *The ptimum temperature* for growth on PDA, SDA and MEA 20–25 °C (Table S1a–c). *Minimum growth* (1–2 mm diam) at 10 °C, and germination of a majority of the conidia at 8 °*C. The maximum temperature* for growth 31 °C (1–3 mm diam). *Keratinolytic activity* weak to moderate (Fig. 10d), with hair attack intensity = 2. *Urease activity* negative (after 14 d of incubation).

*Diagnosis*: *K*. *wagneri* molecularly can be distinguished from other *Keratinophyton* species by ITS locus analysis. Phenotypically, it can be differentiated by combination of the growth rate at 30 °C and conidia size (4.0–8.0 × 2.5–4.0 μm) and morphology (obovoid to clavate, verrucose) (Table 2).

*Additional material examined*: **Slovak Republic**: Tatranská Lomnica, from forest soil, isolated from a different sub-sample, July 2019, *R*. *Labuda*, RL-07 (RL; ITS sequence, MT903275; LSU sequence, MT903309).

*Notes*: Based on a search of the NCBI GenBank nucleotide database, the closest hit for *K. wagneri* using the ITS sequence was *K. minutisporosum* (as *Chrysosporium minutisporosum* CBS 101577; GenBbank: KT155616); with identity = 486/541 (90%) and gaps 11/541 (2%). Morphologically, *K*. *wagneri* can be separated from *C*. *minutisporsoum* by its larger conidia (4.0–8.0 × 2.5–4.0 μm vs. 3.0–4.0 × 1.5–3.5 μm) and growth rate at 25 °C on PDA after 14d (25–30 mm vs. 55–70 mm). *Keratinophyton straussii* and *K. wagneri* seem to be very similar, however, they can be distinguished by: (1) size of conidia (av. = 4.9 × 2.5 μm vs. 5.7 × 3.2 μm), (2) growth at 30 °C on PDA (15–20 mm vs 3–4 mm diam), (3) morphology of conidiogenous cells (commonly vs. non– to occasionally swollen), (4) colony pigmentation on SDA after prolonged incubation (bright orange vs. dark brown), and (5) keratinolytic ability after 3 wk. (very strong vs moderate). In addition, the production of a pinkish pigment on PCA after 3–4 wk. (at 20 °C and 25 °C) has been observed only in *K. wagneri*. Moreover, conidia of this species are more coarsely roughed (warty) than those in *K. straussii* (Fig. 8c–e)*.*

All four new species are readily distinguished from the other taxa in the genus *Keratinophyton*, based on phenotypical characteristics such as growth at high temperature and/or conidial morphology (Cano and Guarro 1990; Cano and Guarro 1994; Currah 1985). The most important species-specific phenotypic distinguishing characteristics are found as morphology of conidia (shape, surface and dimensions) and growth rate at 30 °C after 14 d on PDA.

## KEY TO SPECIES OF *KERATINOPHYTON*

This key is modified from that of Cano et al. (2002). The given data for source and origin represent the type strains of the related species.

**Table:** 

1	Ascomata developed	**2**
	Ascomata not developed	**6**
2 (1)	Ascospores 7.5–8.5 × 4.5–5 μm; from arable soil, Spain	**saturnoideum**
	Ascospores smaller	**3**
3 (2)	Ascospore with broad equatorial rim	**4**
	Ascospore with narrow equatorial rim	**5**
4 (3)	Ascospores discoid; daily growth 3–4 mm at 28 °C on PYE agar and reverse uncoloured; from beach soil, Spain	**hispanicum**
	Ascospores with pitted equatorial rim; cruciform in lateral view; daily growth 2–3 mm at 28 °C on PYE and reverse cream coloured; from soil, Nepal	**durum**
5 (3)	Ascospores lenticular, 5–6 × 2.5–3.5 μm; pronounced radial ridges at 37 °C on PYE agar and reverse uncoloured; from lawn soil, India	**terreum**
	Ascospores with conoid poles, 4–4.5 × 2–2.5 μm; ridges absent at 37 °C on PYE agar and reverse uncoloured; from arable soil, Spain	**punsolae**
6 (1)	No or restricted (<1 cm in diam) growth at 30 °C on PDA; intercalary conidia absent	**7**
	Good growth (> 1cm in diam) at 30 °C on PDA	**10**
7 (6)	Conidia smooth; racquet hyphae present	**8**
	Verrucose conidia; racquet hyphae absent; forest soil, Slovakia	**wagneri**
8 (7)	Conidia obovoid to ellipsoidal, 2.2–4.3 × 1.6–3.2 μm; reverse yellowish on PDA at 25 °C; from soil under the chicken feather, China	**hubeiense**
	Conidia larger	**9**
9 (8)	Conidia clavate to long-ellipsoidal; colony reverse brown in centre and light yellow in margin at 25 °C on PDA; from plant root soil, China	**clavisporum**
	Conidia obovoid to clavate; colony reverse white to slightly yellowish at 25 °C on PDA; from forest soil, Slovakia	**gollerae**
10 (6)	Intercalary conidia absent	**11**
	Intercalary conidia present	**12**
11 (10)	Conidia smooth, ellipsoidal or fusiform; colony reverse white to light yellow at 25 °C on PDA rhizosphere soil, China	**linfenense**
	Conidia verrucose, obovoid to clavate; colony reverse white with slightly yellowish centre at 25 °C on PDA; from garden soil, Italy	**straussii**
12 (10)	Conidia echinulate, obovoid to clavate; colony reverse orange yellow at 25 °C on PDA; from sole of the foot, Czechia	**echinulatum**
	Conidia verrucose	**13**
	Conidia smooth, or smooth to verrucose	**15**
13 (12)	Conidia 3–4 μm wide; colony reverse white at 25 °C on PYE agar; from river sediments, Spain	**minutisporosum**
	Conidia obovoid to clavate; colony reverse different colour than white on PYE agar	**14**
14 (13)	Conidia more than 3 μm wide; colony reverse pale brown at 25 °C on PYE agar; from soil, India	**pannicola**
	Conidia up to 3 μm wide; colony reverse brownish orange at 25 °C on PYE agar; from river sediments, Spain	**fluviale**
15 (12)	Conidia smooth	**16**
	Conidia smooth to verrucose	**18**
16 (15)	Conidia pyriform to oval, 5–7 × 3.5–5 μm; from barber shop soil, India	**turgidum**
	Conidia smaller	**17**
17 (16)	Conidia ellipsoidal, clavate to cylindrical; racquet hyphae absent; colony reverse yellowish at 25 °C on PDA; from farmland soil, China	**qinghaiense**
	Conidia obovate to clavate; racquet hyphae present; colony reverse lemon yellow at 25 °C on PDA; from compost soil, Austria; from compost soil, Austria	**lemmensii**
18 (15)	Conidia cylindrical to clavate, 5–30 × 2–3.5 μm; colony reverse initially uncoloured and later pale brown at 25 °C on PDA; from garden soil, Spain	**siglerae**
	Conidia 4–35 × 2.5–5 μm; colony reverse yellowish white at 25 °C on PDA; from river sediments, Spain	**submersum**

## DISCUSSION

### Phylogeny

Phylogenetic reconstruction using ITS sequences resulted in clustering of a new species, *Keratinophyton lemmensii*, with *K. durum* (as *Aphanoascus durus*; Cano and Guarro 1990)*, K. hubeiense* (as *Chrysosporium hubeiense*; Zhang et al. 2016) and *K. submersum* (as *Chrysosporium submersum*; Vidal et al. 2002), and forming a sister clade to *K. siglerae* (as *Chrysosporium siglerae*; Cano and Guarro 1994)*.* The other three novel species, *K. gollerae*, *K. straussii,* and *K. wagneri,* were resolved in a separate terminal clade (Fig. 1a). Its sister clade encompasses *K. clavisporum* (as *Chrysosporium clavisporum*; Zhang et al. 2017), *K. quinghaense* (as *Chrysosporium quinghaense*; Han et al. 2013), *K. linfenense* (as *Chrysosporium linfenense*; Liang et al. 2009), and *K. turgidum* (Sharma and Shouche 2017). Based on the phylogeny and as a result of the abandoning of separate names for morphs of the same fungus (May et al. 2019), species previously described in *Chrysosporium* require redisposing in the genus *Keratinophyton*. In our study we confirmed ten species required transfer. The monophyletic genus *Keratinophyton* is now extended and includes 25 species including ten species known from sexual morphs (Sutton et al. 2013; and this paper) and 15 species which are currently known only from asexual morphs (including the recently described *K. turgidum* (Sharma and Shouche 2017). The species known only from the asexual morphs can be distinguished by particular combinations of their morphological traits (colony colour and growth rate, growth response at higher/lower temperatures, as well as morphology of conidia) and differences in the ITS regions (Fig. 1a, Table 2).

### Ecology and distribution

Almost all known *Keratinophyton* species have been isolated from soil or soil-like substrates, such as river sediments, compost and sand (Table 1; Cano and Guarro 1990; Sharma and Shouche 2017; Labuda et al. 2008; Liang et al. 2009; van Oorschot 1980; Vidal et al. 2000; Vidal et al. 2002). Hubalek (2000) provided a list of keratinolytic fungi associated with free-living mammals and birds of which *Keratinophyton pannicola* (as *Chrysosporium evolceanui*) has been isolated from a variety of animals, different species of rodents in Australia, Czechia, Germany, the UK, and the former Yugoslavia; a rabbit in Canada; and from birds in Australia (Queensland), Czechia, India, and the former Yugoslavia. *Keratinophyton durum* (as *Aphanoascus durus*) has been isolated from a hedgehog in Ivory Coast, and *K. terreum* (as *Aphanoascus terreus*) has been found associated with a variety of rodents in Czechia, Germany, India, Nigeria, Romania, and the former Yugoslavia and, and further birds in Australia (Queensland) Czechia, India, the USA, and the former Yugoslavia (Hubalek 2000). To the best of our knowledge, there is only a single report of a human clinical isolate belongs to *K. echinulatum* (CCF 4652 = CBS 141178) from the sole of the foot of a 35-year-old woman in Czechia (Hubka et al. 2016). However, Hubka et al. (2016) indicated that the etiological significance of this fungus was unclear, and they concluded that the infection was actually caused by another dermatophyte, which was not isolated or was overgrown by *K. echinulatum*. A few other cases have been published in a small range of animals including *Keratinophyton pannicola* (as *Chrysosporium pannicola*) from skin of a dog in former Yugoslavia (Hajsig et al. 1974; van Oorschot 1980) and from a case of keratomycosis in a horse (Grahn et al. 1993).

In her review on *Chrysosporium* and related genera in *Onygenaceae*, Sigler (2003) stated that some reports concerning *Chrysosporium* species as etiological agents must be viewed with caution, in case the isolated fungus has neither been identified to species level nor documented well enough to confirm the aetiology. In the follow-up list of medically relevant species provided by Sigler (2003), no species is mentioned as being currently affiliated within the genus *Keratinophyton,* while *K*. *pannicola* (as *C*. *pannicola*) is included in the *Atlas of Clinical Fungi* (de Hoog et al. 2020) as a concern in skin infections. Even though the keratinophilic fungi were considered as potential pathogens by several researchers (Rippon 1982; Papini et al. 1998); they rarely cause infections. Therefore, soil is proposed as an epidemiological and probably also an evolutionary link, that relates geophilic, zoophilic, and anthropophilic keratinophilic fungi (Papini et al. 1998). Interestingly, during a mycological investigation of the soil samples in the present study, a high prevalence of geophilic dermatophytes such as *Nannizzia gypsea* from Italy (collected in 2004), a co-occurrence of *Arthroderma uncinatum* with *Aphanoascus keratinophilus* (as *Chrysosporium keratinophilum*) from the Slovak Republic (collected in 2011), and *Arthroderma terrestre* along with abundant *A. uncinatum* from Austria (collected in 2015) were noted (data not shown).

As the members *Keratinophyton* are considered as typical soil-borne fungi (Cano and Guarro 1990; Cano et al. 2002; Sutton et al. 2013) and there is no solid evidence of pathogenicity, it is likely that previously reported animal-associated cases reflect environmental transmissions from soil to the animals during activities in contact with soil. The ability of these fungi to persist and survive in the soil was observed also during the present study, as in case of *K. straussii*, the type strain was isolated 11 years after sampling in 2004, and two more strains (RL-05 and RL-06) representing the same taxon were isolated in a repeated study even 15 years after the sampling. Likewise, a second strain (RL-07) used for the description of *K. wagneri* and the type of *K. gollerae* (BiMM-F250) were both isolated 8 years after the samples were collected.

The degree of keratin degradation by the novel strains described here varied. It was very strong in both *K. gollerae* and *K. straussii* compared to other tested strains, attacking the cuticle and cortex of hairs with about 50–80% degradation. In addition to keratin degradation, keratinolytic fungi share common properties with dermatophytes (Marchisio et al. 1994; Mitola et al. 2002). Even though some of these fungi can grow at 37 °C (Fig. 1a), potential pathogenicity to homeothermic vertebrates (mammals and birds) by these fungi seems highly unlikely because of some presumably missing pathways in their metabolism. Instead, their strong keratinolytic ability might be providing a competitive advantage in the nature to acquire nutrients from hair and may have potential in industry for the production of proteolytic enzymes to degrade keratinous materials (hairs, fur, feathers, etc.). Furthermore, these fungi represent a yet unexplored possible source of new bioactive compounds as there is not much known of these properties in the genus (Kushwaha and Guarro 2000).

## Supplementary Information

**Additional file 1: Table S1a-c.** Temperature dependent growth of the new *Keratinophyton* species (in mm) on PDA, MEA and SDA.

## Data Availability

The phylogenetic trees constructed for the study can be found in TreeBASE, http://purl.org/phylo/treebase/phylows/study/TB2:S28290. The data analysed in this study are also available from the corresponding author on reasonable request.

## Abbreviations

*A*: *Aphanoascus*
BiMM: Bioactive Microbial Metabolites group
*C*: *Chrysosporium*
CBS: Centraalbureau voor Schimmelcultures (in Westerdijk Fungal Biodiversity Institute), CCF Culture Collection of Fungi
CMA: Corn Meal Agar
CTAB: Cetyltrimethyl ammonium bromide
*Ct*: *Ctenomyces*
CZ: Prague
FMR: Facultat de Medicina in Ciències de la Salut
IFO: Institute for Fermentation, Osaka
IMI: International Mycological Institute
ITS: Internal transcribed spacer region
LSU: Nuclear large subunit rDNA
M: Microcolonies
ML: Maximal likelihood
MEA: Malt Extract Agar
NBRC: NITE Biological Resource Center, Japan
nSG: No spore germination
PCR: Polymerase chain reaction
PCA: Potato Carrot Agar
PDA: Potato Dextrose Agar
PRM: Mycological Department, National Museum, Prague, Czech Republic
PYE: Phytone Yeast Extract Agar
rDNA: Ribosomal DNA
RL: Roman Labuda Personal Collection, Research Platform Bioactive Microbial Metabolites
SEM: Scanning electron microscopy
SDA: Sabouraud 4% Dextrose Agar
SG: Spore germination
UAMH: University of Alberta Microfungus Collection and Herbarium
YEW: Water with 2–3 drops of 10% yeast extract
