# Supplementary material for: Molecular systematics of Keratinophyton: the inclusion of species formerly referred to Chrysosporium and description of four new species
Source: IMA Fungus. 2021 Jul 8;12:17. doi: 10.1186/s43008-021-00070-2 (PMC8265132; doi:10.1186/s43008-021-00070-2)
Supplement: Supplementary file 1 — Additional file 1: Table S1a-c. Temperature dependent growth of the new Keratinophyton species (in mm) on PDA, MEA and SDA. [file 43008_2021_70_MOESM1_ESM.docx]

**Table S1 a** Temperature dependent growth of the new *Keratinophyton* species (in mm) on PDA after 14 d.

| Species | Temperature (°C) | | | | | | | | | | | |
| --- | --- | --- | --- | --- | --- | --- | --- | --- | --- | --- | --- | --- |
|  | 8* | 10 | 12 | 15* | 18 | 20 | 25* | 28 | 29 | 30* | 31* | 32 |
| *K. lemmensii* | 1‒2 | 4‒5 | 7‒9 | 10‒14 | - | 25‒27 | 28‒35 | - | - | 38‒45 | 32‒38 | M‒1 |
| *K. gollerae* | SG‒M | M‒1 | 2‒6 | 15‒18 | - | 18‒20 | 20‒22 | 11‒12 | M‒1 | SG | - | - |
| *K. straussii* | nSG | 1‒2 | 4‒5 | 7‒9 | 18-20 | 18‒22 | 24‒28 | - | - | 15‒20 | 6‒8 | 1‒2 |
| *K. wagneri* | SG | 1‒2 | 8‒10 | 6‒8 | 18-20 | 20‒25 | 25‒30 | - | - | 3‒4 | 2‒3 | M‒1 |

*Crucial distinctive growth temperatures; SG, spore germination; nSG, no spore germination; M, microcolonies

**Table S1 b** Temperature dependent growth of the new *Keratinophyton* species (in mm) on MEA after 14 d.

| Species | Temperature (°C) | | | | | | | | | | | |
| --- | --- | --- | --- | --- | --- | --- | --- | --- | --- | --- | --- | --- |
|  | 8* | 10 | 12 | 15 | 18 | 20 | 25 | 28 | 29 | 30* | 31* | 32 |
| *K. lemmensii* | 1‒2 | 2‒4 | 5‒7 | 7‒10 | - | 15‒17 | 20‒25 | - | - | 18‒20 | 12‒15 | SG |
| *K. gollerae* | SG-M | M | 1‒2 | 5‒8 | - | 10‒12 | 14‒16 | M | nSG | - | - | - |
| *K. straussii* | nSG | M | M | 4‒7 | 10‒12 | 10‒12 | 18‒20 | - | - | 5‒10 | 3‒4 | M‒1 |
| *K. wagneri* | SG | M | M‒2 | 3‒5 | 10‒13 | 12‒15 | 18‒20 | - | - | M | M | SG |

*Crucial distinctive growth temperatures; SG, spore germination; nSG, no spore germination; M, microcolonies

**Table S1 c** Temperature dependent growth of the new *Keratinophyton* species (in mm) on SDA after 14 d.

| Species | Temperature (°C) | | | | | | | | | | | |
| --- | --- | --- | --- | --- | --- | --- | --- | --- | --- | --- | --- | --- |
|  | 8* | 10 | 12 | 15* | 18 | 20 | 25 | 28 | 29 | 30* | 31* | 32 |
| *K. lemmensii* | 1‒2 | 6‒7 | 8‒10 | 7‒10 | - | 24‒26 | 28‒35 | - | - | 30‒32 | 25‒30 | M‒1 |
| *K. gollerae* | SG‒M | M‒1 | M-1 | 20‒22 | - | 12‒17 | 23‒25 | M‒2 | SG | SG | nSG | - |
| *K. straussii* | nSG | M‒1 | 2‒3 | 3‒4 | - | 14‒16 | 16‒20 | - | - | 15‒20 | 1‒2 | nSG |
| *K. wagneri* | SG | M‒1 | 1‒3 | 3‒4 | - | 12‒14 | 14‒18 | - | - | M‒1 | SG‒M | nSG |

*Crucial distinctive growth temperatures; SG, spore germination; nSG, no spore germination; M, microcolonies
